# Profiling Enzyme Activity of l-Asparaginase II by NMR-Based Methyl Fingerprinting at Natural Abundance

**DOI:** 10.1021/jacs.3c02154

**Published:** 2023-05-08

**Authors:** Rachayita Nag, Srishti Joshi, Anurag Singh Rathore, Subhabrata Majumder

**Affiliations:** † Biophysics & Structural Genomics, Saha Institute of Nuclear Physics, Kolkata 700064, India; ‡ Department of Chemical Engineering, Indian Institute of Technology, Hauz Khas, New Delhi 110016, India; § Homi Bhabha National Institute, Anushaktinagar, Mumbai 400094, India

## Abstract

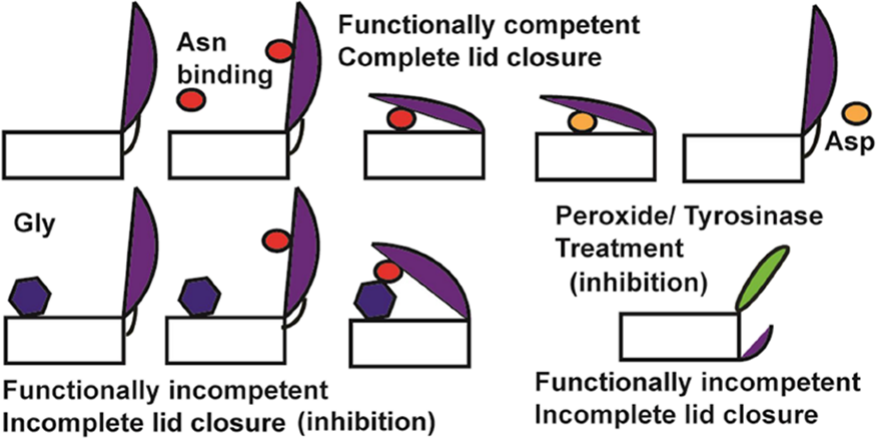

l-asparaginase II (MW 135 kDa) from *E. coli* is an FDA-approved protein drug used for the treatment of childhood leukemia. Despite its long history as a chemotherapeutic, the structural basis of enzyme action, in solution, remains widely contested. In this work, methyl-based 2D [^1^H-^13^C]-heteronuclear single-quantum correlation (HSQC) NMR, at natural abundance, has been used to profile the enzymatic activity of the commercially available enzyme drug. The [^1^H-^13^C]-HSQC NMR spectra of the protein reveal the role of a flexible loop segment in the activity of the enzyme, in solution. Addition of asparagine to the protein results in distinct conformational changes of the loop that could be signatures of intermediates formed in the catalytic reaction. To this end, an isothermal titration calorimetry (ITC)-based assay has been developed to measure the enzymatic reaction enthalpy, as a marker for its activity. Combining both ITC and NMR, it was shown that the disruption of the protein conformation can result in the loss of function. The scope, robustness, and validity of the loop fingerprints in relation to enzyme activity have been tested under different solution conditions. Overall, our results indicate that 2D NMR can be used reliably to gauge the structure–function of this enzyme, bypassing the need to label the protein. Such natural abundant NMR methods can be potentially extended to probe the structure–function aspects of high-molecular-weight protein therapeutics (glycosylated protein drugs, enzymes, therapeutic monoclonal antibodies, antibody–drug conjugates, and Fc-fusion proteins), where (a) flexible loops are required for their function and (b) isotope labeling may not be straightforward.

## Introduction

The enzyme *E. coli*l-asparaginase ( l-asparaginase amidohydrolase, EC 3.5.1.1) is an FDA-approved drug for the treatment of pediatric acute lymphoblastic leukemia.^1^ In addition, the drug has demonstrated activity against acute lymphoblastic leukemia and lymphosarcoma non-Hodgkin’s lymphoma.^2,3^ Recently, the drug has been shown to retard the metastasis of breast cancer tissue as well.^4^ The mechanism of drug action relies on the enzymatic conversion of asparagine to aspartate, thereby depleting the pool of bioavailable asparagine in serum for the tumor cells. Unlike normal cells, tumor cells lack the asparagine synthetase enzyme and cannot synthesize the critical nutrient, asparagine, *de novo.* Hence, the use of the l-asparaginase enzyme deprives the tumor cells of its critical nutrients and leads to cell death.

Despite its use in chemotherapy, the structural basis of enzyme action remains widely contested.^5−8^ The structure of the free enzyme has been determined for wild-type enzymes (3ECA.pdb, 1NNS.pdb, and 6V23.pdb).^9^ The functional form of the protein is a homo-tetramer with an approximate molecular weight of 135 kDa. Residues Thr12, Tyr25, Thr89, Asp90, and Lys162 are presumed to be the active sites of the enzyme.^7,9,10^

**Figure 1:**
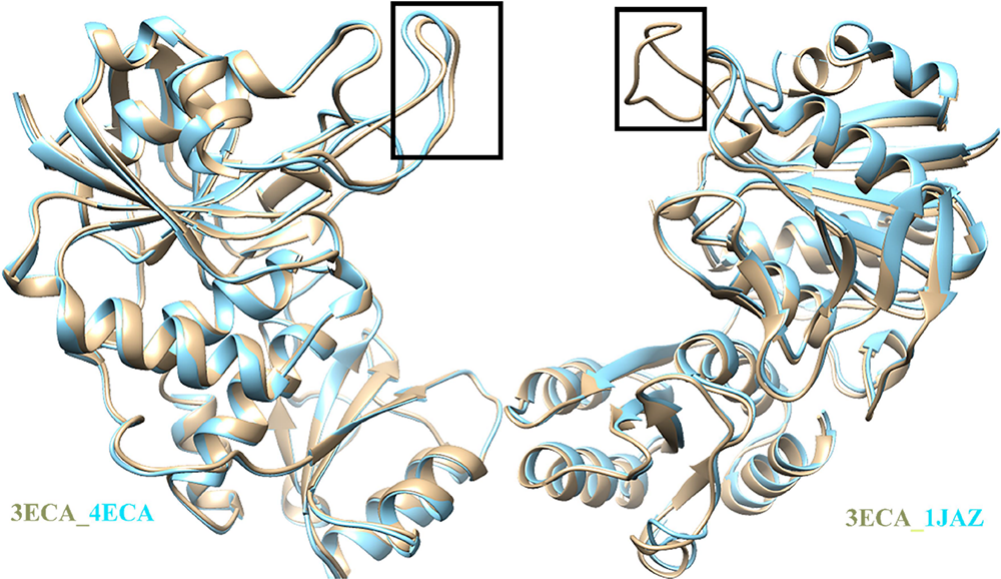
Superposition of the crystal structures of l-asparaginase II shows a high degree of overall similarity. The lid loop residues (marked by the box) are well resolved in the wild type (3ECA.pdb) and T89V mutant (4ECA.pdb) but not in D90E mutant (1JAZ.pdb). In addition, deviations in the loop can be observed between 3ECA.pdb and 4ECA.pdb, suggesting differential flexibility of the loop residues across the crystal structures of the enzyme.

An intriguing feature of the crystal structure(s) is a lid loop segment between amino acids 10–30, which bears the catalytic residues T12 and N24 (Figure 1). In the crystal structure of the free enzyme, this particular segment is generally not resolved (1JAZ.pdb), unless the protein gets co-crystallized with the product aspartate (3ECA.pdb and 1NNS.pdb).^6,9,11^ This suggests that the loop segment could be inherently flexible and the presence of aspartate may be required to adopt a well-resolved conformation.^12^ The conformation(s) of the mobile loop and its solvent exposure can be potentially correlated with the susceptibility of the enzyme toward its proteolytic cleavage in blood serum.^13^ To this end, Asn24 has been identified as the primary cleavage site for both asparagine endopeptidase and cathepsin B from site-directed mutagenesis studies.^14^ The role of dynamics of the mobile loop, in the enzyme action, has also been implicated in the stopped flow and fluorescence studies of the double mutant W66Y/Y25W.^15^ An overlay of the free enzyme (3ECA.pdb) and asparagine-bound mimic/intermediate (4ECA.pdb) shows (a) a high degree of overall similarity (backbone RMSD = 0.34 Å) in structure and (b) higher *B* values of Tyr 25 and Val 27 in the latter, suggesting higher flexibility in these residues in the bound state.^7^ The degree of spatial flexibility of the loop can be difficult to gauge from the different crystal structures due to the presence of active site mutations, that is, D90E (1NNS.pdb) or T89V (3ECA.pdb). The deviation of C^β^ of the loop residues between 3ECA.pdb and 4ECA.pdb also does not reveal any abrupt changes, except for the mutation site (A27V) (Table S1). Moreover, crystal packing forces and pH-induced artifact make the interpretation of dynamics of loop residues in catalysis less obvious. Furthermore, QM/MM studies of wild-type enzyme-based 3ECA.pdb also outlay the critical role of T12 and Y25 in the mobile loop in attaining the correct conformation required for the transition state of the reaction.^8^ While the role of the loop residue T12 is known to be covalently participating in the enzyme reaction, the conformation of the mobile loop in free form, or its plausible changes, in the presence of the substrate remains largely unknown. Moreover, the preferential dynamics of loop residues for l-asparaginase have been correlated with substrate specificity.^16^ Hence, there appears to be a missing link in understanding the role of the mobile lid loop in the function of l-asparaginase, despite the availability of high-resolution crystal structures.

Protein loop conformations are often critical to their function.^17,18^ The conformational dynamics of the active site loops, in the cases of enzymes, have been studied extensively by NMR.^19−23^ Functional enzyme dynamics in millisecond-to-second timescales, at the residue level, have been quantitatively studied by solution NMR, especially using relaxation dispersion experiments.^23−26^ Such studies require the assignment of amino acid residues to be known *a priori*. An earlier NMR study of l-asparaginase II using ^13^C-/^15^N-/^2^H-labeled samples, however, reported missing backbone amide assignment for around 20% of protein residues, including the functionally important loop residues (BMRB 27588).^27^ This limits the use of 2D [^15^N-^1^H] HSQC NMR in probing lid loop conformation(s) in solution. However, methyl-bearing residues, owing to favorable NMR relaxation properties, are often excellent probes of structure/dynamics of high-molecular-weight proteins and protein assemblies.^28,29^ While the spatial description of the dynamics may not be derived from the NMR data alone, qualitative insights into the role of the loop dynamics in the enzyme function can be potentially obtained from this methyl fingerprinting. Such fingerprinting at natural abundance can be potentially complementary to the rigorous quantitative temporal dynamics of the proteins using labeled amino acids and relaxation-based NMR methods. Finally, the enzymatic activity of the protein asparaginase is often measured by coupled enzymatic assay using colorimetry.^30^ These methods are indirect readouts of enzyme activity and may suffer from secondary interferences. Hence, we posit that structural fingerprints of the loop, if obtained, from high-resolution NMR methods, can be used as a direct and robust readout for enzyme activity. This is relevant especially if the role of the loop conformation is critical in enzyme activity.

To test our hypothesis, we applied 2D [^1^H-^13^C] HSQC NMR, in natural abundance, on the commercially available wild-type l-asparaginase II drug in the presence and absence of asparagine. Under the experimental conditions, cross-peaks corresponding to the loop residues of l-asparaginase II were observed in the intact protein spectra. The mobile loop conformation of the protein was identified in solution for the first time, to the best of our knowledge. The addition of asparagine induced distinct changes in the loop conformation, as evident from the NMR spectra. In order to connect the changes of the loop conformation with the enzyme activity, apparent reaction enthalpy was also determined from isothermal titration calorimetry (ITC). The reliability of the combined biophysical assay in predicting the enzyme activity was tested with the (a) use of glycine and (b) chemical modification of the loop residue tyrosine (Y25). Finally, this method was also applied to probe the impact of methionine oxidation, if any, on the activity of the enzyme.

## Materials and Methods

The commercial l-asparaginase II, Bionase 5000 IU (Zydus , India) available as lyophilized powder, was used throughout the study. Monosodium hydrogen phosphate, disodium hydrogen phosphate, glycerol (99.5%, molecular biology grade), and boric acid were purchased from Sisco Research Laboratories Pvt., Ltd. (Mumbai, India). l-Asn, d-Gly, d-Asp, l-Asp, and mushroom tyrosinase K were purchased from Sigma Aldrich (St. Louis, MO, USA). The peptide corresponding to the lid loop GGTIAGGGDSATKSNYTAGKVG, used in the study, was purchased from Creative Biolabs (Shirley, New York, United States). All buffers were prepared fresh and used within 2 weeks of preparation. The plasmid pET28a encoding the wild-type sequence of N term 6X-His-tagged l-asparaginase was obtained from Genscript (USA).

### Concentration Measurement for NMR and ITC

One vial of lyophilized l-asparaginase (Bionase 5000 IU) was dissolved in 2 mL of 100 mM sodium phosphate buffer at pH 7.8. Excipients were removed by extensive dialysis against the same buffer (2 L) at least four times. The sample was then concentrated to 500 μL, using an Amicon filter (MWCO 3 kDa, Thermo-Fisher). The sample concentration was checked by a Nanodrop spectrophotometer using the molar absorptivity E1% value of 8.099. For NMR studies, 110 μM (or more) enzyme concentration is used. For ITC experiments, serial dilutions were made from the same stock. Lastly, a glycerol buffer (36% v/v) of 145 μM enzyme, in the presence and absence of equimolar Asn, was also used for NMR. The glycerol buffer sample was not used for ITC.

### NMR Data Acquisition and Processing

All NMR data were recorded on an 800 MHz Bruker Advance III spectrometer equipped with triple resonance TCI room-temperature probes with a triple axis gradient system. ^13^C methyl fingerprint data were collected at 40 °C. Unless otherwise noted, 800 MHz ^1^H-^13^C correlation data sets were recorded with 160 scans per transient and 160 × 2048 complex points corresponding to spectral widths of 40 ppm × 14 ppm, with acquisition times of 9 and 90 ms in the *t*_1_ (^13^C) and *t*_2_ (^1^H) domains, respectively. The ^1^H carrier was placed on water resonance, and the ^13^C carrier was set to 21 ppm. A recycle delay of 1.2 s was employed for phase-sensitive improved-sensitivity heteronuclear single-quantum coherence experiments (hsqcetgpsi). The spectra of asparaginase in glycerol buffer in the presence and absence of Asn were acquired at 50 °C. Identical acquisition parameters were used for all the peptides except for the intact loop peptide where 160 scans were used at 45 °C. For peptides P4, P5, P6, P7, and P8, 80 scans were used at 40 °C. For peptides P1, P2, and P3, 80 scans were used at 25 °C. Tyrosinase-treated peptide was run at 40 °C with NS = 40. NMR data were processed in Topspin 3.6.2 software. The processed file was read into CARA software, where peak picking and peak intensities were determined for subsequent analysis. [^1^H-^13^C] ALSOFAST-HMQC spectra were recorded at 800 MHz (with RT probe) with 1.024 scans and 226 × 2048 complex points, corresponding to spectral widths of 29.8 × 19.5 ppm with acquisition times of 18 and 65 ms in the *t*_1_ (^13^C) and *t*_2_ (^1^H) domains, respectively.^31−33^ For this purpose, Topspin 4.1.4 version was used which has an in-built pulse program “afhmqcgpphsf” in the Bruker library. The ^1^H and ^13^C carriers were placed on water resonance and at 20 ppm. A recycle delay of 0.4 s was applied. ReBurp selective pulse on the resonance of methyl groups with a bandwidth of 29.0 ppm was used for selective refocusing of methyl resonances. The two pulse schemes for [^1^H-^13^C] ALSOFAST-HMQC and [^1^H-^13^C] HSQC NMR experiments for the same sample were performed with identical acquisition parameters, as listed in Tables S2 and S3. All ALSOFAST-HMQC spectra were processed in Topspin 4.1.4 and were visualized in CARA software. The peak intensities were calculated using volume integrals and the base rectangle sum integration method in Cara, using a peak width of 0.005 ppm. The spectral similarities were gauged by the use of linear regression plot of intensities of the peaks across the set of acquired spectrum for the samples.

### Assignment of the Loop Residues of the Protein

The use of enzyme, at natural abundance, limits the sensitivity of conventional triple resonance heteronuclear NMR methods for residue assignment. ^1^H-^1^H TOCSY and ^1^H-^1^H NOESY have been used in view of the segmental mobility of the loop but remain of limited use^34^ (data not shown). In order to assign the methyl residues of the loop, several truncated fragments of the peptide were used to systematically identify the methyl cross-peaks belonging to each residue. The list of peptides used are shown in Table 1.

**Table 1:** List of Peptides Used for the Assignment of Lid Loop of l-asparaginase (T12-**V30**)

peptide number	sequences of amino acids
intact peptide	GGTIAGGGDSATKSNYTAGKVG
peptide 1	GGGIAGGGDSATKSNYTAGKVG
peptide 2	GGGDSATKSNYGAGKVG
peptide 3	GGGDSTKSNYGAGKVG
peptide 4	GGGDSATKSNYTAGKVG
peptide 5	GGGDSATKSNYTAGKG
peptide 6	GGGDSAKSNYTAGKVG
peptide 7	GGTIAGGG
peptide 8	GGTIGGG

The assignments of the protein were obtained by transferring the assignments of the peptide to the protein, as shown in Figure S1. First, the assignment of methyl peaks of the intact peptide was attempted. To assign **T12**, the intact peptide spectra were superimposed with that of peptide 1. As isoleucine methyl occupies a distinct region of the spectra, the superposition of peptide 1 and peptide 2 allowed for the assignment of **I13**. The superimposition of peptide 7 with peptide 8 enabled the assignment of residue **A14**. Assignment of **A20** was made by the superposition of the spectra of peptides 2 and 3. Similarly, the assignment of **T26** was obtained from the superimposition of the spectra of peptides 2 and 4. Assignment of **V30** was obtained by comparing the spectra of peptides 4 and 5. Superimposition of peptides 5 and 6 led to the assignment of **T21**. Assignment of **A27** was obtained from the superimposition of peptides 7 and 8. All the spectra were compared against each other to validate the correctness of the residue assignment. The workflow of assignments is summarized in Table S4. In our workflow, HSQC was chosen in order to focus on highly flexible regions, thus simplifying the NMR spectra and resonance assignment.

### Activity Assay by Isothermal Titration Calorimetry

The l-asparaginase II samples made as above were used for ITC. l-Asn was dissolved in 100 mM sodium phosphate buffer to prevent any buffer mismatch. The activity assay was developed on ITC Microcal 200 (Malvern PA, USA) in a way that the reaction enthalpy does not saturate the heater feedback. Additionally, the reaction could be repeated to see the experimental variation of reaction enthalpy upon successive injections. The optimum reaction conditions were found to be 10 nM of enzyme in the cell and 10 mM L-Asn in the syringe. The default cell volume was 280 μL. The injection volume was optimized to 3 μL with a spacing of 600 s between each injection. Initial delay after baseline equilibrium was set at 180 s. All the ITC experiments were conducted at 25 °C. The stirring speed was set to be 400 cycles per second. The initial power level was set at 10 dB. With each 3 μL injection, the concentration of the substrate (asparagine) in the cell becomes 0.1 mM compared to 10 nM enzyme in the cell. The above ITC parameters were used for all the samples. In order to check the robustness of the assay and for assessing the biophysical stability of asparaginase, a freshly prepared enzyme solution was stored at 4 °C. The ITC titration was performed at the end of 60 days to validate the activity of the enzyme before and after the storage period.

### ITC Data Analysis

Data analysis was done with the built-in Origin software (Originlab Corporation, Northampton, MA). The raw data (thermogram) is a plot of change of power with time. Each point in the plot denotes a power level at that time. Generally, the initial power is the same as the one that is specified in the experimental design, that is, 10 dB. At the point of injection, there is a significant change in the power level (*p*) because of the initiation of the reaction. The reaction continues until the substrate asparagine completely converts into product aspartate, and the power comes back to the basal level again. Power is represented by d*q*/d*t*, the rate of change of heat. Therefore, the integration of power over time gives us heat (expressed in calorie) in one injection. The area under the curve gives the heat of reaction *Q*.
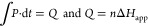
Here, *n* is the number of moles of asparagine converted. Hence, the observed reaction heat is a measure of extent of reaction, that is, the number of moles of asparagine hydrolyzed at a given time. For successive injections, the reaction enthalpy is also determined. Since *n* is known (asparagine syringe concentration) and is constant in all experiments, Δ*H*_app_ measures the extent of the reaction. When the ITC experimental parameters like the amount of asparaginase in the cell, amount of asparagine in the syringe, and interval between injections are all kept constant, the measured apparent enthalpy provides an estimate of the enzyme activity.

### Inhibition of Asparaginase Activity by Glycine

It has been previously reported that glycine can inhibit the action of l-asparaginase in vivo.^35^ To this end, the inhibition of l-asparaginase activity by glycine was studied by ITC. This titration was conducted with the same experimental parameters as mentioned before, except for the 10 mM (Gly + L-Asn) solution in the syringe. The ITC experimental parameters were identical to those of l-Asn addition experiments.

### Tyrosinase Assay

Enzymatic modification of amino acids can provide insights into the structure function relationship of proteins. Tyrosinase K is known to modify the exposed tyrosine residues in the protein and l-asparaginase II in particular and has caused a loss of activity.^36^ Since the mobile loop of l-asparaginase contains Tyr 25, it may undergo modification upon tyrosinase K treatment. The commercial tyrosinase enzyme was dissolved in 50 mM potassium phosphate buffer, pH 6.5, and stored in −20 °C. To this end, the enzymatic assay was optimized for ITC and NMR experiments. After optimization, 115 μM of Bionase and 57.5 μM of tyrosinase K (in 2:1 ratio) were kept in 50 mM sodium phosphate buffer at 25 °C for 8 days. The extent of total tyrosine modification was monitored from UV–visible measurements at 280, 300, and 350 nm. As a control, 115 μM of Bionase was also used without tyrosinase under identical conditions. Samples were aliquoted for UV–vis spectroscopy and ITC at 0, 1, 4, and 8 days. The extent of modification was measured from the UV–vis spectra, and the enzymatic activity was determined from ITC. The corresponding structural changes were probed with 2D NMR on day 4.

### Impact of Hydrogen Peroxide by NMR

Oxidation of proteins has been known to alter the structure and dynamics of proteins.^37,38^ For oxidation studies with H_2_O_2_, EMPLURA, hydrogen peroxide––30% (MERCK) was used. Stock solutions of 0.5 and 0.1% H2O2 were made by appropriate dilutions of 30% H_2_O_2_. Two lyophilized Bionase 5 K vials were reconstituted with the above two solutions, respectively. The vials were incubated at 25 °C for 14 h. The H_2_O_2_-treated asparaginase solutions were buffer-exchanged in the same 100 mM sodium phosphate buffer (pH 7.8) by dialyzing extensively to remove extra H_2_O_2_ present in the protein solution. The dialyzed proteins were concentrated using an Amicon 3 kDa MWCO filter. The protein was concentrated to 1 mL, having a concentration of 95 μM. 10 mM asparagine stock solution was prepared by dissolving 1.32 mg of l-asparagine in 1 mL of sodium phosphate buffer, pH 7.8.

### Detection of Methionine Oxidation by RP-HPLC

#### Concentration Measurement and Sample Preparation

Lyophilized samples were reconstituted in deionized water (MilliQ), and the protein concentration was determined by UV absorbance spectroscopy at 280 nm (Nanodrop 2000, Thermo Fisher Scientific, Waltham, Massachusetts, United States). For HPLC experiments, the lyophilized product was dissolved in deionized water to a concentration of 1 mg/mL, carefully filtered through a 0.2 μM syringe filter, transferred to a HPLC glass high-recovery vial (Agilent Technologies, Santa Clara, California, United States), and the required volume was injected into the HPLC column (Dionex UltiMate 3000 RSLC Systems, Thermo Fisher Scientific, Waltham, Massachusetts, United States).

#### Peroxide-Induced Oxidation Assay

To understand the impact of oxidation on the structure, reconstituted l-asparaginase was incubated with varying concentrations of H_2_O_2_ for a period of time and analyzed through reverse-phase liquid chromatography (RPLC). For RPLC-based experiments 50 μg of the enzyme was incubated with 0, 0.1, 1, and 10% H_2_O_2_ (v/v) for 14 h at 10 °C. Thereafter, the samples were centrifuged briefly at 10,000 rpm for 2 min to sediment any particles, and the supernatant was carefully transferred into a high-recovery high-performance LC (HPLC) vial. 3 μg of each sample (0, 0.1, 1, and 10% H_2_O_2_) was injected into the column (Zorbax 300 SB-C8 stable bond analytical, 5 μm, 4.6 × 150 mm, Agilent Technologies, Santa Clara, California, United States) connected to an ultra-HPLC (UHPLC) system (Dionex Ultimate 3000 RSLC system, Thermo Scientific, Waltham, Massachusetts, United States), operated at 25 °C. The sample components were separated over a 30 min linear gradient (mobile phase A: 0.1% trifluoroacetic acid––TFA in deionized water; mobile phase B: 0.1% TFA in acetonitrile) at a constant flow rate of 1 mL/min. Detection was performed by monitoring UV absorbance at 280 nm.

#### Colorimetric Assay for the Enzymatic Activity of l-Asparaginase

The performance of ITC-based activity assays of l-asparaginase was also compared with that of the colorimetric assay of the enzyme. For the colorimetric assay, Nessler’s reagent was used. In the activity assays, different reagent concentrations were used, as shown in Table S5. For example, a combination of 107 μM Asn and 10 nM enzyme was chosen to mimic the ITC-like condition (condition 1). Other conditions include a combination of 1 mM Asn and 1 μM asparaginase (condition 2). In all conditions, the absorbance at 436 nm was recorded in a Nanodrop spectrophotometer.

#### Site-Directed Mutagenesis

The site-directed mutagenesis was performed using the In-Fusion mutagenesis kit of Takara Bio, USA, Inc., using the manufacturer’s protocol. The primer sequences of the forward and reverse primers were:

Forward––*5’TGGTAAAGGGGGCGTTGAGAACCTGGTGAAC3’.*

*Reverse––5’ ACGCCCCCTTTACCAACGGTGTAGTTGCTCTTG3’.*

Briefly, following PCR amplification/linearization, the PCR product was purified using a PCR clean-up kit by Qiagen Inc. The consecutive DNA ligation step was performed using the Takara In-Fusion snap assembly mix. The sequencing of the generated DNA was carried out using T7 forward and T7 reverse primers from *Eurofins Genomics India Pvt., Ltd*. Since the plasmid contained N-terminal 6× His tags, nickel affinity chromatography (Qiagen Inc) was performed to purify the wild-type and V30G mutants. Both the proteins were semipurified, and the activity assay using colorimetry was performed.

## Results

### Mobility of the Lid Loop from NMR Spectroscopy

**Figure 2:**
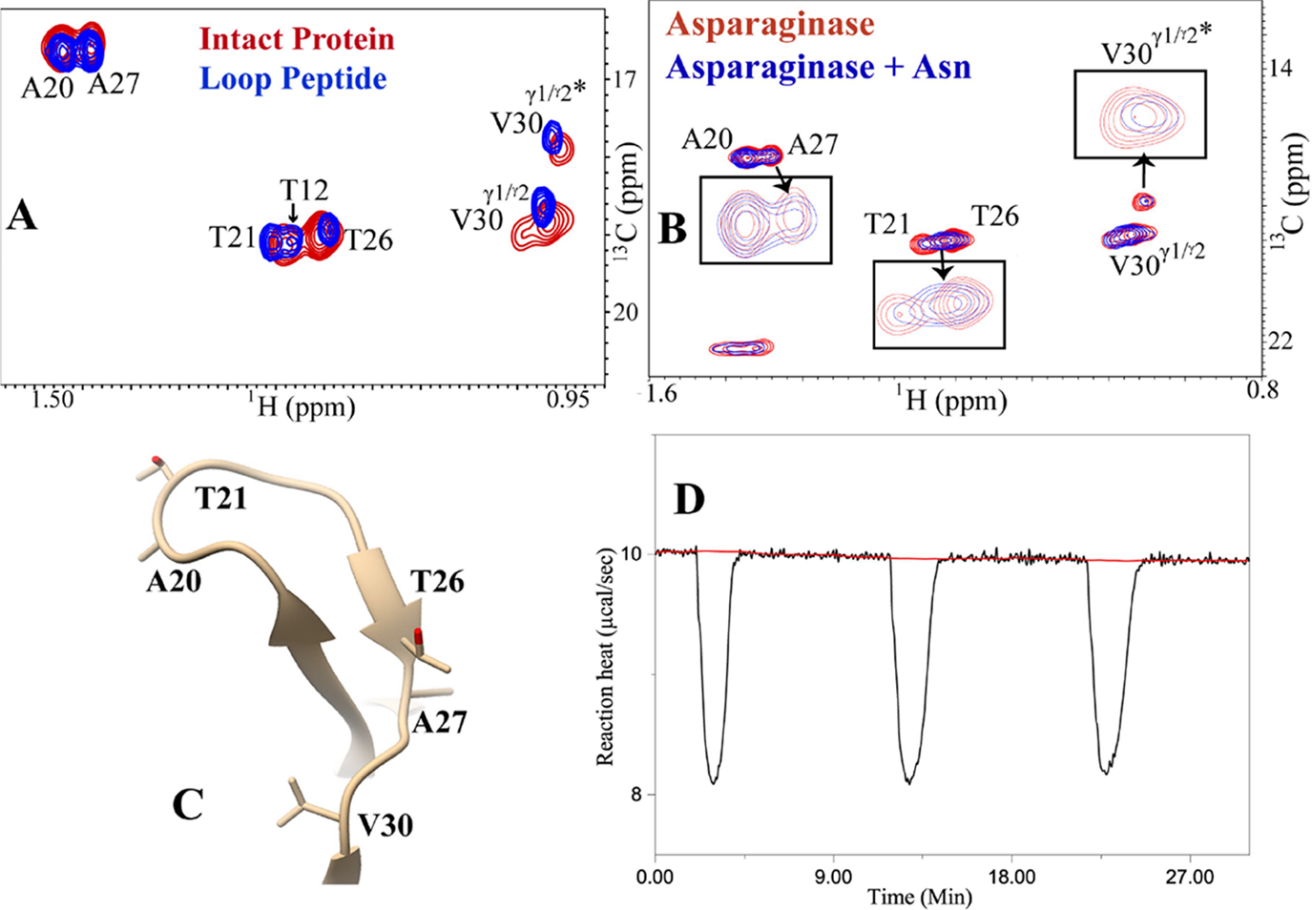
Critical role of loop residues in enzymatic catalysis performed by l-asparaginase. (A) Overlay of 2D [^1^H-^13^C] HSQC NMR spectra of wild-type lasparaginase protein (red) with the protein’s loop peptide (blue) shows similar peak disposition. The cross-peaks corresponding to methyl-bearing residues are assigned. The peak corresponding to T12 in the loop is broadened in the intact protein. Similarly, the I13 peak is also broadened in the intact protein. (B) Overlay of 2D [^1^H-^13^C] HSQC of asparaginase in the presence (red) and absence of asparagine (blue) is shown. The addition of the substrate l-Asn (1:1 ratio) to the enzyme induces specific peak broadening in **T21**, **T26**, **A27**, and **V30**. The peaks with significant broadening are highlighted in squared insets. (C) Residues are mapped onto the crystal structure of l-asparaginase (3ECA.pdb). (D) Thermogram showing the conversion of L-asparagine to L-aspartic acid by l-asparaginase II, obtained from isothermal titration calorimetry. At the beginning of the titration, the baseline (black solid line) remains at the basal level (indicated by red solid line), at the dB value ∼10. With the initiation of the enzymatic reaction following the first injection, the baseline drifts below the 10 dB value and returns to the initial value, indicating the complete conversion of asparagine to aspartate. The area under the curve gives us the reaction heat, Δ*H*_app_ ∼ −5.4 kcal/mol. For all ITC experiments, 10 nM of the enzyme was kept in the cell, and a stock solution of 10 mM Asn was kept in the syringe. Repetition of the injections (3 μL) allows for gauging the reproducibility of the reaction heat and hence can be a measure of enzymatic activity.

Methyl-bearing amino acids (I, L, V, T, and M) can be used as molecular probes of structure and dynamics of high-molecular-weight proteins in solution NMR.^39^ A selectively protonated ^13^C methyl group in a deuterated background enables the acquisition of [^1^H, ^13^C] HMQC spectra for proteins up to 1 MDa. Under natural abundance, such methyl fingerprinting has been used for the structural characterization of monoclonal antibodies.^32,40^ In this work, the commercial protein drug, l-asparaginase (MW 135 kDa) has been used at natural abundance. Despite the high molecular weight and unfavorable transverse relaxation, the protein yields a set of well-resolved peaks in the methyl region of the [^1^H-^13^C] HSQC spectrum. This suggests that the peaks may belong to residues in the flexible segment(s) of the protein. The lid loop region (10aa–30aa) harbors six glycine residues, which may result in an increased flexibility. An overlay of the [^1^H-^13^C] HSQC spectrum of the intact loop peptide with that of the intact protein suggests that the peaks from the intact protein likely correspond to those of mobile loop residues (Figure 2A). The peaks were assigned following the scheme shown in Table S4. While there is an overall similarity in the disposition of peaks between the loop peptide and that of protein, key differences were observed. Unlike in the peptide, I13 (^δ^CH3) peaks are broadened out in the intact protein (Figure S2). In the case of peptide, the Ile side-chain χ_2_ dihedral angle can sample four distinct conformations in solutions (trans and gauche−). The chemical shift of the isoleucine methyl group, in the peptide, suggests that the rotameric distribution of trans (δCH3 = 14.8 ppm) and 85% gauche (−) conformations is sampled by the free peptide.^41^ Even in the peptide, one of the peaks corresponding to I13 ^δ^C is broadened at a high temperature (Figure S2). Similarly, T12 is also broadened out in the protein, unlike in the peptide (Figure 2A). For **V30**, the observation of ^γ1^C and ^γ2^C chemical shifts suggests the presence of a wide range of rotameric states (gauche (+), gauche (−), and trans) in slow-exchange timescales.^42^ Such a distribution is preserved both in the isolated peptide and the intact protein for **V30** (Figure 2A). Thus, although the loop peptide has the same primary sequence, differences exist in their respective conformational states when it is part of the protein, possibly driven by the scaffolding effect of the protein.^18^

### Involvement of the Mobile Loop in Catalysis

It is known that Tyr 25 and T12 of the mobile loop are involved in the catalysis of l-asparaginase.^5^ Distinct changes were noted in the free enzyme spectra when L-asparagine (200 μM) was added at 1:1 concentration (Figure 2B). This contrasts the kinetic assay conditions, where a typically excess substrate concentration relative to the protein is used. In our work, protein concentrations were optimized such that the (a) intermediate(s) formed during catalysis can be detected, (b) sufficient concentration of protein is used for detection by NMR, and (c) substrate asparagine concentration is above the Michaelis Menten constant (*K*_m_) of asparagine (11.5 μM).^9^ A distinct broadening in the residues **T21** and **T26** was noted (Figure 2B). In addition, one of the ^γ1^C peaks corresponding to **V30**, that is, V30* is also broadened. The correlation plot of methyl peak intensities of asparaginase in the presence and absence of asparagine indicated an overall correlation of 0.5, suggesting changes in the loop conformation (Figure S3A). Moreover, the residues **T21**, **T26**, V30*, and **A27** appear as outliers in the correlation plot (Figure S3A). These specific spectral changes are unique signatures for the conformational changes of the loop. Unlike L-asparagine addition, when d-aspartate is added (200 μM) to the protein in (1:1) ratio, no such changes were noted, and the overall correlation coefficient is 0.96 (Figure S3B). To rule out any experimental artifacts, the spectra of the free enzyme was acquired in duplicate. The methyl fingerprints are nearly identical with the overall correlation coefficient of 0.97 (Figure S3C), suggesting a high degree of spectral similarity (Figure S3B,C). L-Asparagine addition-induced spectral changes are indicative of the presence of conformational state(s) during the enzyme catalysis. Among many such states, the asparagine-bound enzyme could be one of the conformational states. The affected loop residues are mapped on to the structure, as shown in Figure 2C.

### Enthalpy Profiling of l-Asparaginase Activity

In this work, ITC has been used to measure the reaction heat when l-asparagine is enzymatically converted to l-aspartate. A single injection provides the apparent reaction heat when excess L-asparagine is added to the enzyme. The apparent reaction enthalpy (Δ*H*_app_) is −5.4 kcal/mol. Repetition of the injections also demonstrate the reproducibility of Δ*H*_app_ across the ITC injections (Figure 2D). Reproducibility of the enzyme assay has been tested by using asparaginase samples stored at 4 °C over 2 months (data not shown).

### Inhibition of Enzyme Activity by Glycine

**Figure 3:**
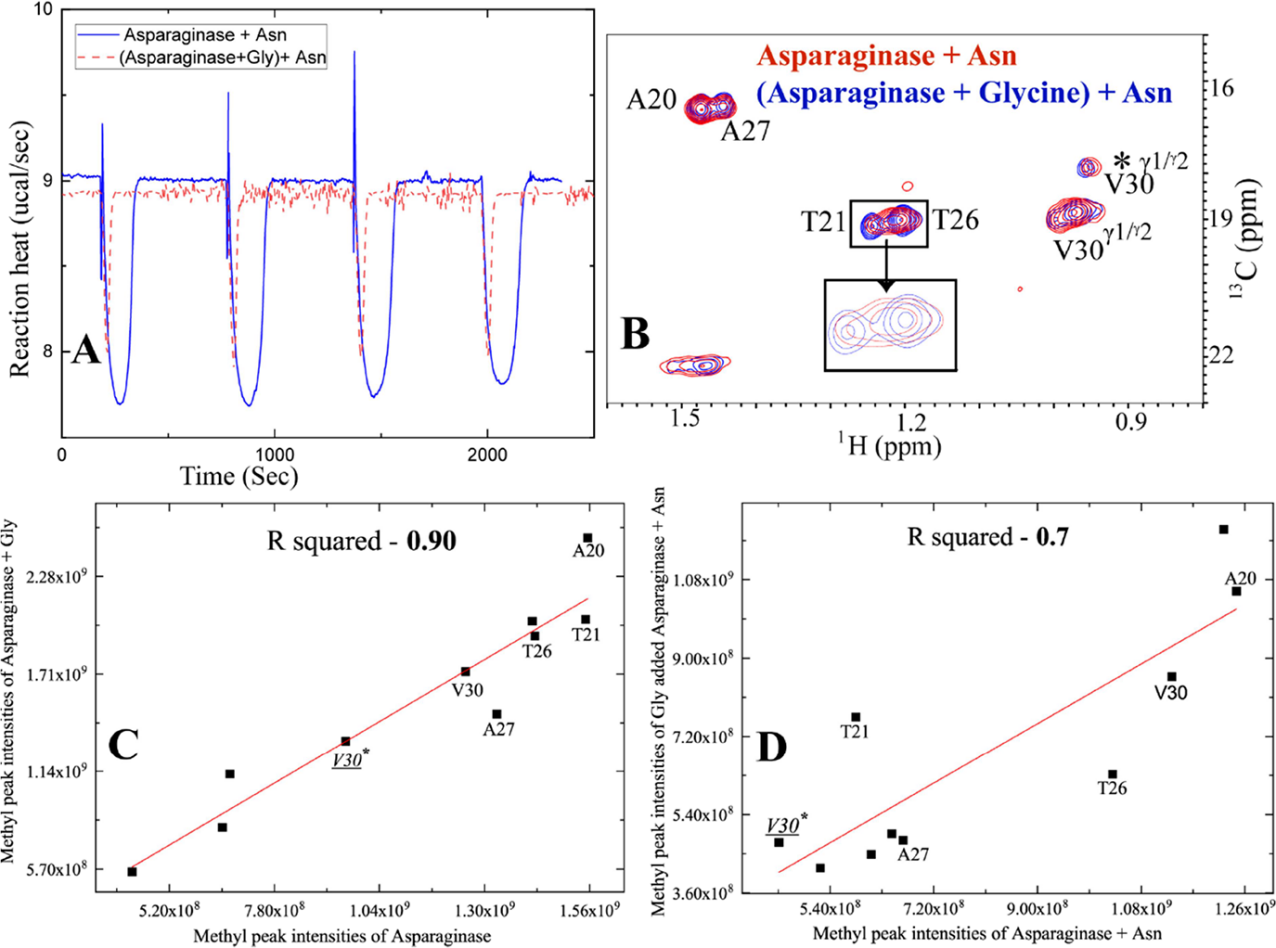
Glycine inhibits the enzyme activity of l-asparaginase II without perturbing the mobile loop. (A) Addition of glycine inhibits the enzymatic conversion of asparagine by l-asparaginase II, as evident from ITC. In ITC, 10 nM of the enzyme was kept in the cell, and a stock solution of either 10 mM Asn or (Asn + Gly) was kept in the syringe. At each injection, 3 μL of the substrate/substrate mixture was used. A comparison of the thermograms shows the catalysis of l-Asn to Asp in the absence (red) and presence of Gly (red). The area under the curve, due to Asn + Gly addition, is much less, which indicates a lesser reaction heat or apparent reaction enthalpy ∼ −1.04 kcal/mol. This suggests that the enzymatic activity of l-asparaginase (i.e., no. of moles of Asn hydrolyzed per unit time) is lost in the presence of glycine. (B) Overlay of 2D [^1^H-^13^C] HSQC NMR spectra of asparaginase (+Asn) (red) with asparaginase and glycine (+Asn) (blue) shows no significant broadening of the critical binding residues (**T21**, **T26**, **A27**, and **V30**) of the loop. The absence of spectral changes due to Asn addition in l-asparaginase is highlighted in the signature of the inset. The molar ratio of the enzyme, glycine, and Asn is 1:1:1 for the NMR experiments only. (C) Linear regression plot between the methyl peak intensities obtained from the 2D [^13^C-^1^H] HSQC NMR spectra of asparaginase vs glycine-added asparaginase infers both the spectra as nearly identical (*R*^2^ = 0.9). The intensities for all the peaks (assigned) are shown in Table S10. (D) Linear regression plot between the methyl peak intensities obtained from the 2D [^1^H-^13^C] HSQC NMR spectra of asparaginase in the presence of l-Asn vs asparaginase (+Gly) in the presence of l-Asn gives a correlation coefficient of 0.7. Thus, the extent of broadening in these residues of asparaginase, upon Asn addition, is different when glycine is present.

ITC and methyl NMR were used to probe the impact of glycine on the enzymatic activity of l-asparaginase. The addition of 107 μM (Asn + Gly) to 10 nM asparaginase (both dissolved in 100 mM sodium phosphate buffer, pH 7.8) in every injection (3 μL volume), shows a much less reaction heat of −1047 cal, compared to that of the addition of 107 μM Asn (−5673 cal) (Figure 3A). This suggests that the enzymatic activity is altered in the presence of glycine.

The mechanistic basis of glycine inhibition of l-asparaginase was probed by methyl-based fingerprinting of the loop. Enzyme inhibition could, in principle, be achieved either by (a) perturbing the loop conformation or (b) by preventing the loop to attain the catalytically competent conformation in the transition/intermediate state.^5^ However, when asparagine was added to a solution containing equimolar amounts of asparaginase and glycine, no changes in peak residues **T21**, **T26 V30**, or **A27** were observed, suggesting that the catalysis may not proceed (Figure 3B). On the other hand, examination of the asparaginase spectra in the presence and absence of glycine reveals no significant differences, as evident from the overall correlation coefficient of 0.90 (Figures S3D and S4). This suggests that glycine either binds very weakly (*K*_m_ −7 mM for l-asparaginase) or does not bind at all.^43^ Alternatively, binding of glycine may not impact the lid loop at all. The spectral dissimilarity of asparaginase in the presence of Gly + Asn, and Asn alone, is evident in the correlation plot shown in Figure 3D. The use of natural abundance asparaginase limits the sensitivity of detection of nonloop residues that may be binding to glycine and may be potentially important for catalysis. This shows that the perturbation of loop residues in the free enzyme may not be a necessary condition for the loss of activity.

### Robustness of the Method

#### Tyrosinase Treatment of the Enzyme

**Figure 4:**
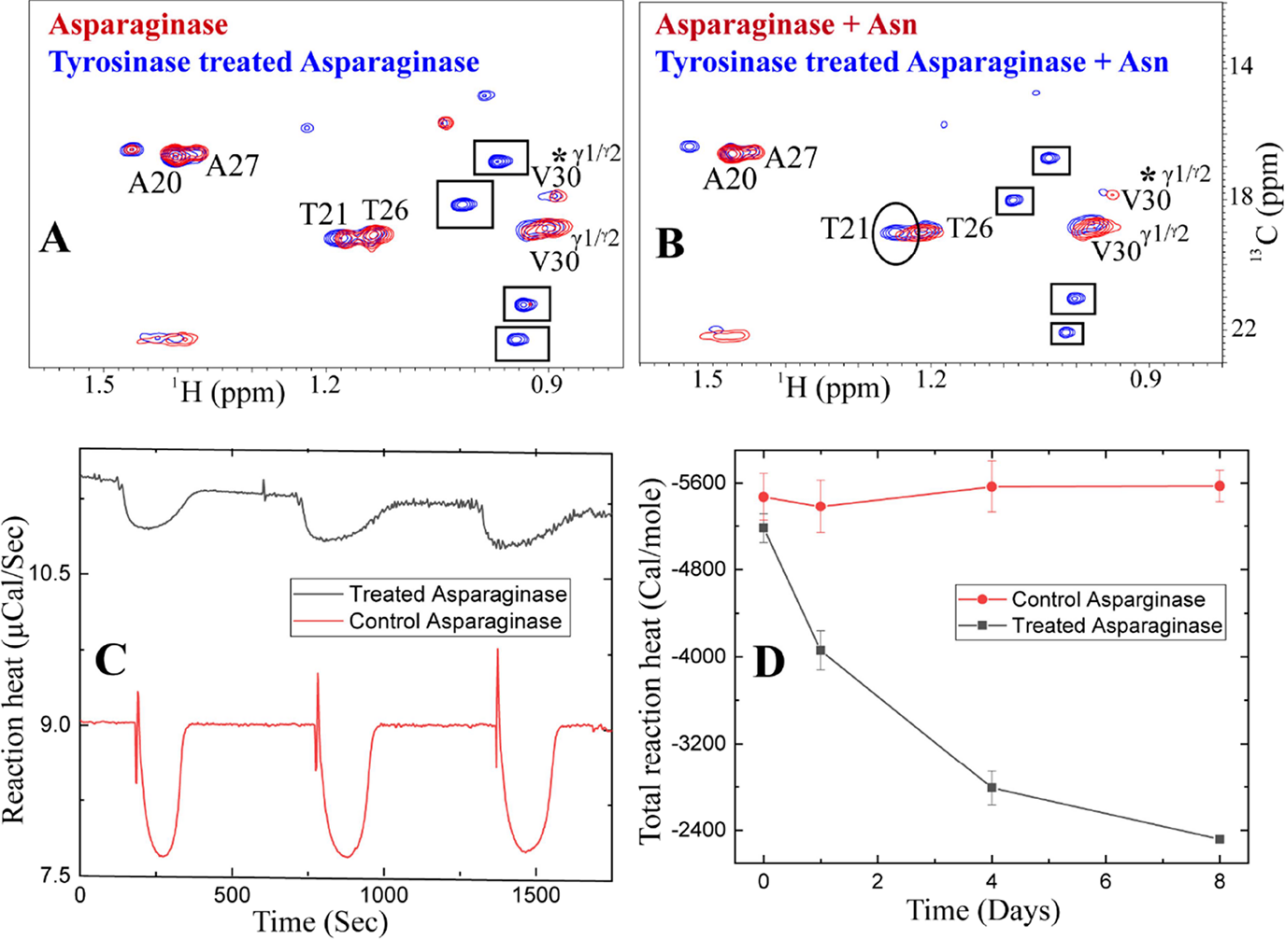
Mushroom tyrosinase K treatment results in the loss of asparaginase activity. (A) Overlay of 2D [^1^H-^13^C] HSQC spectra of 110 μM asparaginase (red) with tyrosinase (57 μM)-treated asparaginase (110 μM) (*T* = 4 days) (in blue) shows the appearance of many new peaks that were not present in any of the spectra acquired before and are highlighted in squares. The presence of new peaks suggests the conformational change of l-asparaginase due to tyrosinase treatment. (B) Overlay of 2D [^1^H-^13^C] HSQC spectra of asparaginase in the presence of asparagine (red) with tyrosinase-treated asparaginase in the presence of asparagine (blue) shows no broadening of peaks upon substrate addition. The new peaks are not impacted due to Asn addition as well. (C) Tyrosinase treatment of asparaginase at *T* = 4 days significantly lowers Δ*H*_app_ (*T* = 4 days) ∼ −2.7 kcal/mol. (D) Reaction heat of asparagine conversion decreases over time. For example, Δ*H*_app_ of tyrosinase-treated l-asparaginase (black) decreases from ∼ – 5.6 kcal/mole (*T* = 0 day) to −2.7 kcal/mol (*T* = 4 days) to −2.3 kcal/mol (*T* = 8 days). On the other hand, untreated asparaginase (red) retains similar Δ*H*_app_ during this period. For all ITC experiments, 10 nM of the enzyme was kept in the cell, and a stock solution of 10 mM L-Asn was kept in the syringe.

The robustness of the NMR-based structure–function assay has been tested under different conditions. To rationalize the sanctity of loop conformation in relation to enzyme activity, l-asparaginase was treated with mushroom tyrosinase K. Since the loop contains Tyr 25, it could be prone to oxidation by tyrosinase treatment. Monitoring of UV absorbance at 280, 300, and 350 nm suggested that tyrosine oxidation has indeed happened due to the increase in absorption at 300 nm (0.87 to 1.44) and 350 nm (0.52 to 0.93), whereas the same for control samples was 0.63 to 0.88 and 0.42 to 0.59, respectively, over a period of 4 days. (Figure S5-A). Samples at *T* = 4 days were used for NMR. Indeed, the methyl fingerprints of tyrosinase-treated l-asparaginase samples suggest distinct conformational changes in the protein (Figure 4). New peaks marked with rectangle were observed in the overlay of the [^1^H-^13^C] HSQC spectra of tyrosinase-treated l-asparaginase with that of free asparaginase (Figure 4A). The presence of new cross-peaks suggests that tyrosinase treatment induced distinct conformational changes in the protein in slow-exchange timescales. The addition of L-asparagine to tyrosinase-treated asparaginase does not induce broadening in residue **T21**, as evident in the case of untreated asparaginase (Figure 4B). Clearly, the methyl fingerprints of tyrosinase-treated asparaginase in the presence of L-asparagine suggest that the activity of the protein could have been altered due to tyrosinase treatment.

To corroborate methyl fingerprints with enzyme activity, ITC on tyrosinase-treated samples was performed. The activity of the enzyme drops progressively over time, as is evident from the drop in reaction heat over 8 days from ∼ −5.2 to −2.3 kcal/mol (Figure 4C,D). The raw thermogram of the control l-asparaginase and tyrosinase-treated asparaginase (4 days) also demonstrates this effect (Figure 4C). In order to see whether tyrosine 25 oxidation was sufficient for the change in conformation of the loop, the chemically synthesized peptide corresponding to the loop segment was treated with tyrosinase K. The resulting [^1^H-^13^C] HSQC of the treated peptide demonstrates that the treated peptide does not show the peaks (indicated by rectangle) that appeared in the tyrosinase-treated protein (Figure S5B), and the loop peptide fingerprint remains unaltered (Figure S5C). This suggests that the tyrosinase-induced changes in the protein may or may not be limited to the loop. Inspection of the tyrosine oxidation pathway reveals the formation of intermediate quinones which are strong oxidizing agents, capable of oxidizing cysteine or methionine residues in the protein.^44^ The standard redox potential for the two-electron reduction of dimethyl sulfoxide is +160 mV while that for cystine is +220 mV.^45^ Cys 77 and Cys 105 in l-asparaginase are involved in disulfide linkages. Among the methionine residues, Met 121 is located spatially closer to the loop segment (distance ∼3.8 Å), the oxidation of which can potentially perturb the loop conformation, resulting in the loss of activity (Table S6). The chemically synthesized loop segment does not have any methionine and is not expected to demonstrate the conformational change akin to the protein. Finally, the overlay of [^1^H-^13^C] spectra of free asparaginase with that of tyrosinase alone eliminates the possibility of the new peaks to be coming from tyrosinase K itself (Figure S5D). Last but not the least, the extent of spectral dissimilarity due to the tyrosinase treatment of l-asparaginase was also evident from the methyl correlation plots, the correlation coefficient of free asparaginase versus tyrosinase-treated asparaginase being 0.54 (Figure S6A). Furthermore, the correlation coefficient of tyrosinase-treated asparaginase spectra in the absence and presence of Asn is 0.74 (Figure S6B).

#### Hydrogen Peroxide Treatment of the Enzyme

**Figure 5:**
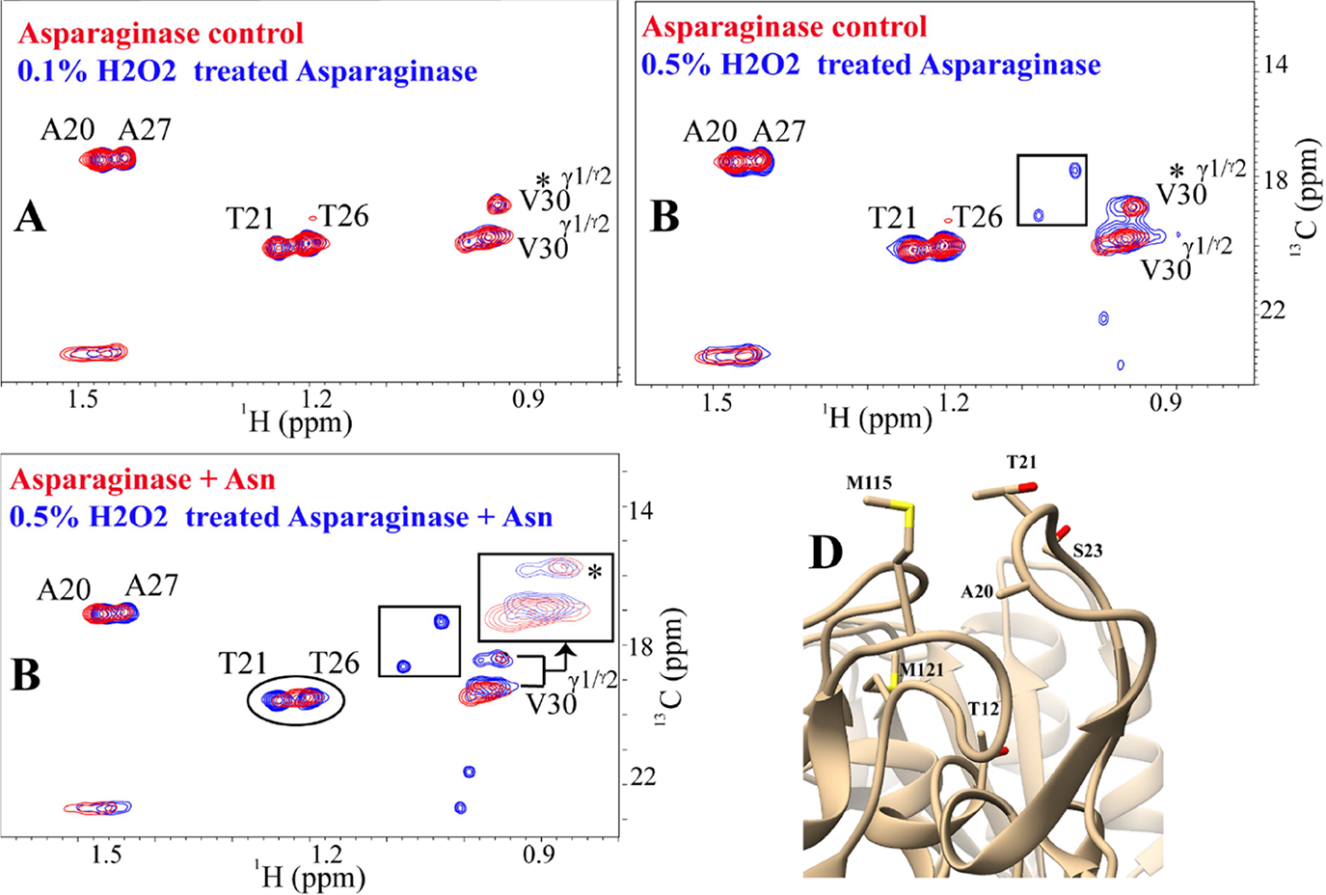
H_2_O_2–_mediated oxidation of l-asparaginase alters the conformation of l-asparaginase and results in the loss of enzyme activity. (A) Overlay of 2D [^1^H-^13^C] HSQC spectra of asparaginase in the presence (blue) and absence (red) of 0.1% H_2_O_2_ is shown, which reveals no change in spectra. (B) Overlay of 2D [^1^H-^13^C] HSQC spectra of asparaginase in the presence (blue) and absence (red) of 0.5% H_2_O_2_ is shown, which shows the presence of new peaks. (C) Overlay of 2D [^1^H-^13^C] HSQC spectra of 0.5% H_2_O_2_-treated asparaginase in the presence of Asn (blue) and control asparaginase in the presence of Asn (red) is shown. For the 0.5% H_2_O_2_ treatment, no peak broadening at **T21**, **T26**, and **V30** was observed, unlike the untreated one. This suggests that the conformational change of the loop required for catalysis could not be achieved for a 0.5% H_2_O_2_-treated sample, resulting in the loss of enzyme activity, similar to tyrosinase treatment. (D) Plausible site of oxidation, that is, the methionine residues M 121 is located close to **T26** of the lid loop (∼3.8 A). Hence, the formation of methionine sulfoxide may potentially alter the loop conformation and hence disrupt the enzymatic activity.

To study the role of methionine oxidation in the disruption of loop conformation, l-asparaginase was treated with 0.1% H_2_O_2_ and 0.5% H_2_O_2_. The 0.1% H_2_O_2_-treated asparaginase does not show any differences in methyl fingerprinting as compared to the free protein (Figure 5A). However, when the spectral overlay was performed with the free enzyme and 0.5% H_2_O_2_-treated asparaginase, new peaks were observed, suggesting the perturbation of the protein structure (or the loop) (Figure 5B). Differential peak broadening for **T26**, **T21**, **A27**, or **V30** was also not observed due to Asn addition to 0.5% H_2_O_2_-treated asparaginase spectra (Figure 5C). The spectral similarity was retained, as evident from the very high correlation coefficient of 0.91 (Figure S7A). This clearly suggests that the activity of the enzyme has been compromised with 0.5% H_2_O_2_ treatment. The [^1^H-^13^C] HSQC spectra of 0.5% H_2_O_2_l-asparaginse show the presence of new peaks in addition to those of loop residues with comparable intensities. Some of these new peaks overlay with those of tyrosinase-treated peaks, clearly showing that methionine oxidation-induced conformational changes may have occurred during the tyrosinase treatment as well. (Figure S7B). The proof of methionine oxidation was gauged from RPLC.^46^ RPLC utilizes a nonpolar stationary phase and a polar mobile phase to resolve compounds based on their surface hydrophobicity. This rationale was exploited to distinguish the presence of l-asparaginase species with altered surface hydrophobicity generated in response to H_2_O_2_ incubation. Protein oxidation, specifically methionine oxidation, leads to the formation of oxidation polar moieties, such as methionine sulfoxide, that result in decreased surface hydrophobicity. Indeed, an impact of increasing concentration of H_2_O_2_ (0, 0.1, 1, and 10% v/v, 25 °C, 14 h) was observed in the form of generation of multiple species with decreasing hydrophobicity in comparison to the untreated control sample, as evidenced by an earlier elution in the reverse-phase chromatographic profile (Figure S8). Overall, five distinct species were resolved (P1–P5), including the primary species (P1, 11.38 min) in the control sample (Figure S8). At the lowest H_2_O_2_ concentration (0.1% v/v), the retention time (RT) of P1 was slightly left-shifted (P1*, 11.36 min) in the presence of another minor peak to the left (P2, 11.25 min). In the sample incubated with 1% H_2_O_2_ (v/v), the absence of primary species, along with the presence of four lesser hydrophobic species, including P2 was observed. For samples incubated at the highest H_2_O_2_ concentration (10% v/v) species, P2–P4 were absent, and only P5 (10.73 min) was detectable, suggesting the complete oxidation of the sample.

### Limitations of the HSQC-Enabled Methyl Fingerprinting

The spectral data obtained by [^1^H-^13^C] HSQC experiment are sparse and can be potentially improved by using the more recent ALSOFAST-HMQC [^1^H-^13^C] experiments.^32,33^ This is especially relevant for the glycine-mediated inhibition of l-asparaginase, where glycine-induced changes in the l-asparaginase spectra are minimal (Figures S3D and S4). To this end, [^1^H-^13^C] ALSOFAST −HMQC spectra were obtained for asparaginase under various solution conditions. These include the reconstitution of the commercial drug in (a) water (b) in the presence and absence of Asn in sodium phosphate buffer pH 7.8, and in the presence of Gly and Asn (Table S3). A systematic comparison of the two pulse schemes on identical samples reveals the following trends:

(a) ALSOFAST HMQC has a higher S/N ratio compared to HSQC experiments, although the experimental time for the former is nearly half, regardless of the sample conditions (as evident from the increased number of peaks in the spectra). In addition, ALSOFAST HMQC suppresses the nonmethyl peak intensities, which are otherwise present in the HSQC experiment as indicated (Figure S9A,B). However, HSQC has a higher sensitivity for the flexible lid loop residues which can be identified from the overlaid methyl spectra (ALSOFAST HMQC, HSQC) of the protein and the peptide (Figure S9C) and Table S8.
(b) Such a sensitivity gain for ALSOFAST HMQC can be reconstituted in the HSQC spectra by increasing either the temperature or concentration (Figure S10A,B). The additional sensitivity gain, as evident from the increase in the peaks in the leucine/valine region of the spectra, is sensitive for the structured part/non lid loop of the protein.
(c) ALSOFAST HMQC has a lower sensitivity in detecting Asn-induced changes of the lid loop, as evident from the correlation coefficient of 0.7, compared to that of HSQC (Figure S11).
(d) ALSOFAST HMQC clearly differentiates asparaginase conformation in the presence of Asn versus Gly + Asn conditions, as evident from the subsets of peaks that are unique to different substrate/ligand conditions (Figure S12 and Table S9). Last but not the least, ALSOFAST HMQC experiments are ideal for samples containing excipients, since excipient signals (nonmethyl) are completely suppressed in the methyl-selective ALSOFAST HMQC.

### Comparison of Enthalpy-Based Activity Assay with that of Colorimetric Assay

Nessler’s reagent-based assays are used to gauge the enzymatic activity of l-asparaginase. In the colorimetric assay, visible color change was observed only at high enzyme (1 μM) and high substrate (1 mM) concentrations. In ITC-relevant conditions, there was no change in the color between the blank and enzyme samples. There was no color change for condition 2 where an excess substrate (1 mM) compared to enzyme (10 nM) was used. The absorbance at 436 nm for these samples also confirms the above observation (Figure S13A). Thus, while the enthalpy change corresponding to 107 μM substrate and 10 nM enzyme clearly demonstrates the conversion of asparagine to aspartate, colorimetric assays remain unresponsive. In this regard, ITC-based activity assays clearly outperformed the colorimetric assays under the very low substrate condition. Obviously, in the high substrate concentration regime (1 mM), ITC-based assays could not be used because of the generation of excess heat (saturation of signal) (Figure S13B).

## Discussion

### How Does Our Results Compare to the Existing Literature?

In this work, NMR-based methyl fingerprinting of the mobile lid loop of l-asparaginase has been related to its enzymatic activity, as evidenced by the reaction heat. Such an approach enables us to detect the lid loop conformation in a substrate-free state in solution and the possible changes in the conformation in the transition state, albeit qualitatively, through peak broadening in NMR spectra. Such conformational changes are difficult to extrapolate from the currently available X-ray structures of the free enzyme. For example, the crystal structure of commercial l**-**asparaginase 3ECA.pdb (co-crystallized with l-aspartate) suggests that l-aspartate is located nearest to the lid loop residue A27 within a distance of 4.32 Å (Table S7A). Indeed, the [^1^H-^13^C] HSQC spectra of l**-**asparaginase in the presence of l**-**aspartate shows specific peak broadening at A20, A27, and V30 and the modification of residue T26 as well (Figure S14A). On the other hand, the catalytic reaction mode described by the spectral changes of l**-**asparaginase in the presence of l**-**asparagine is unique. This is different from either (a) the methyl fingerprints of the free enzyme or (b) enzyme in the presence of l-aspartate/d-aspartate demonstrated in Figure S14B,C. Thus, methyl NMR fingerprinting of the enzyme under various solution conditions allows us to determine the sanctity of (a) substrate-free loop conformation and the (b) possible intermediate or transition-state conformations. These conformations may be in a quasi-equilibrium condition, so as to be detectable by methyl fingerprinting. The kinetics of the enzymatic reaction, which employs steady-state approximation, remain out of scope of this study. The proof of conformational dynamics (temporal) of the mobile lid loop has been evident from the rapid decrease of the fluorescence emission intensity of wild type enzyme compared to Y25W/W66Y mutant with a half-time of a few milliseconds in the stopped-flow mixing of enzyme and l**-**asparagine.^15^ In the methyl NMR fingerprinting, l**-**asparaginase shows the peak broadening of residue **T26** in the presence of asparagine, which may be due to conformational changes in the immediate vicinity of Y25, in the transition state. Moreover, peak broadening of **T26** may be due to the altered interaction of P117 and Y25 in the transition state, since the presence of interaction can be critical for lid loop opening (Figure S15 and Table S7B). The latter may be responsible for the substrate specificity of l**-**asparaginase.^47^ Moreover, QM/MD simulations on the wild-type enzyme suggest a specific spatial orientation of T12, Y25, and E283 in one of the transition states (first step) of catalysis.^8^ The attainment of the transition-state conformation for the protein may be disrupted in the presence of glycine, as is evident from the overlay of [^1^H-^13^C] ALSOFAST HMQC spectra of l-asparaginase in the presence of Asn + Gly and Asn only (Figure S12C). Thus, the addition of glycine may have disrupted the loop conformation of the transition state, resulting in the loss of enzymatic activity. In this regard, it is important to note that the peak corresponding to the catalytic loop residue T12 is absent both in the absence and presence of Asn. Hence, the spectral data cannot confirm the formation of any stable covalent intermediate formation involving T12 in the wild-type protein, as may be evident in the T89V mutant.^8,7^ While methyl HSQC of l-asparaginase reports on flexible lid loop residues, ALSOFAST HMQC also reports on the non-lid loop residues. This is evident from the additional peaks in the leucine/valine methyl region of the ALSOFAST HMQC spectra. These peaks are severely broadened due to slow rotational correlation times, indicating that they belonged to the structured segments of the protein. The use of ALSOFAST HMQC suggests a definite subset of structured non-lid loop residues that are broadened with (a) glycine addition, (b) Asn + Gly addition, and (c) Asn addition (Figure S12 and Table S9). Clearly, there is a role of non-lid loop residues in the enzyme catalysis beyond the lid loop. These residues may be important for substrate binding or in attaining intermediate state(s), if any. However, the sparseness of the spectral data and the absence of methyl assignments on these residues do not allow deciphering the mechanistic details of enzyme action in its entirety.

### Foray into Enzymatic Dynamics

Enzymes exhibit protein dynamics across a wide range of timescales and magnitudes.^28,29^ Such dynamic states may be difficult to be probed spectroscopically due to the short-lived nature of these states. In this regard, relaxation dispersion-based NMR methods (CPMG) and CEST NMR allow to quantitatively probe the kinetics of interconversion of ground-state conformation with the short-lived excited states, interconverting in s–ms timescales.^25,26^ Literature studies indicate that, through conformational dynamics, enzymes can sample active and inactive conformations,^48^ while in some other cases substrate-free and substrate-bound-like states.^17,23,24^ In these cases, the minor conformational states are short-lived and remain “invisible” by conventional NMR methods. Unlike these rigorous and quantitative assessments, methyl NMR fingerprinting offers a qualitative picture of the functional enzyme dynamics. First, such fingerprinting enables the identification of the catalytically competent conformation of the lid loop in the free enzyme, in solution. The catalytically competent lid loop conformation(s) can be characterized by the presence of (μs–ms) broadening of residues of the protein (T12, I13, and A14) in the absence of the substrate asparagine. The broadening of lid loop residues, upon Asn treatment, may correspond to the presence of multiple conformational states (excited state/transition state) along the reaction trajectory of the enzyme.^49^ The residue-specific dynamics of proteins can be potentially slowed down in the presence of glycerol.^50,51^Indeed, the presence of glycerol causes an inhomogeneous broadening of the lid loop residues (**T21** and **T26**) of free l-asparaginase in glycerol buffer which are otherwise absent in the buffer (Figure S16A,B). This may suggest that alternate lid loop conformations may be stabilized by glycerol affecting the enzymatic activity, as have been reported for enzymes.^52^ These conformations are in slow-exchange timescales. The addition of Asn in glycerol buffer induces peak broadening in **T21** and **T26**, as evident from the spectral overlay. However, the spectral changes do not match with those obtained in the case of Asn addition in buffer (Figure S16C,D). Similarly, it is possible that the presence of both asparagine and glycine stabilizes a catalytically incompetent transition state of the loop Figure S12. Second, tyrosinase treatment or peroxide treatment in buffer stabilizes a catalytically incompetent conformational state of the free enzyme (ground state) that can be directly read out by methyl fingerprinting. Thus, our minimalist approach using the commercial drug protein at natural abundance shows snapshots of the conformational landscapes of the enzyme. The apparent reaction enthalpy from ITC allows to tag such states as catalytically active or inactive. Table 2 summarizes the catalytic mode and noncatalytic mode based on the impact of critical loop residues and the extent of spectral similarity.

**Table 2:** Correlation of Spectral Changes for a Set of Loop Residues of l**-**Asparaginase with the Catalytic Mode of the Enzyme

experimental conditions employed	mode (as obtained from ITC)	relative broadening of intensities (as obtained from NMR)				overall correlation coefficient (*R*^2^) with respect to free enzyme (degree of spectral similarity)
		**T21**	**T26**	**A27**	V30, V30*	
free enzyme						
**(+)** Asn	**catalytic**	**√**	**√**	**√**	**√**	0.5
**(+)** glycine	noncatalytic	×	×	×	×	0.9
**(+)** glycine + Asn	noncatalytic	×	×	√	√	
**(+)** tyrosinase	noncatalytic	×	×	×	√	0.54
**(+)** tyrosinase + Asn	noncatalytic	×	×	×	√	
**(+)** 0.1% H_2_O_2_	noncatalytic	×	×	×	√	0.83
**(+)** 0.5% H_2_O_2_	noncatalytic	×	×	×	√	0.14
**(+)** 0.5% H_2_O_2_ + Asn	noncatalytic	×	×	×	√	

In this regard, the relative contribution of each loop residue in the catalysis is difficult to be determined because of the complexity of enzymatic reaction step(s) and the presence of multiple conformation(s). A closer inspection of the ratio of peak intensities of the two valine 30 peaks supports this hypothesis. The **V30** peak ratio can serve as a marker of rotameric distribution of valine.^42,53^Whenever there is an enzymatic reaction, the ratio of the peak intensities increases upon Asn addition (Table S11). Loss of activity (upon tyrosinase, hydrogen peroxide treatment, or glycine), correlates with the decrease in the ratio. Increase of temperature from 40 to 45 °C may increase the enzymatic activity and is correlated with the increase in the **V30** peak ratio. Similarly, the presence of glycerol may lower the enzymatic activity, which is correlated with the decrease in the **V30** peak intensity ratio. The change in the **V30** peak ratio may indicate the alteration of its rotameric state in catalytically active mode (+Asn). This can be potentially validated from the structure 4ECA.pdb. A superimposition of the free enzyme (3ECA.pdb) and the substrate-bound mimic (4ECA.pdb) suggests changes in the relative orientation of the **V30** side chain. While all the valines (e.g., V45) obey an eclipse orientation in the superimposed structure, **V30** remains in gauche conformation (Figure S17). It may be possible that the **V30** conformational change may also alter the enzyme kinetics much like Y25. Literature evidence suggests that the Y25F mutant is active, although Y25 is a critical residue in catalysis.^54^ In our case, colorimetric assay shows **V30G** mutant is catalytically active (data not shown). Hence, it is possible that **V30** may be involved in substrate binding but not in the overall catalysis. In the spectral analysis, it is important to note that the **V30** peak ratio and the overall correlation coefficient (free vs Asn present) can be used predictably to assess the enzymatic activity of l**-**asparaginase upon Asn addition (Tables 3 and S11).

**Table 3:** Use of Correlation Coefficient to Assess Spectral Similarity

spectral comparison (Asn addition)	correlation coefficient	spectral comparison (addition of ligand/storage)	correlation coefficient	spectral comparison (enzyme treatment)	correlation coefficient
free asparaginase vs asparaginase +Asn at 45 °C	0.24	free asparaginase vs asparaginase + d-aspartic acid	0.96	asparaginase vs tyrosinase-treated asparaginase	0.54
free asparaginase vs asparaginase + Asn at 40 °C	0.50	free asparaginase vs asparaginase + glycine	0.90	asparaginase vs 0.1% H_2_O_2_-treated asparaginase	0.83
asparaginase + Asn vs (asparaginase + glycine) + Asn	0.70	**free asparaginase vs 2 months’ stored asparaginase	0.97	asparaginase vs 0.5% H_2_O_2_-treated asparaginase	0.14
tyrosinase-treated asparaginase vs tyrosinase-treated asparaginase + Asn	0.74				
0.5% H_2_O_2_-treated asparaginase vs 0.5% H_2_O_2_-treated asparaginase + Asn	0.91				
*free asparaginase vs asparaginase + Asn: ALSOFAST	0.70				

Thus, a model for enzymatic activity and its loss can be summarized in Figure S18. The loop conformation can be potentially altered by inducing the chemical modification of amino acid residues, as in the case of tyrosinase or hydrogen peroxide treatment. Since methionine M121 is present in close proximity with **T21**, the oxidation of methionine to methionine sulfoxide may sterically hinder the loop conformation (Figure 5D and Table S6). Similarly, catalytically important T89, although invisible in the HSQC spectra of the protein, is located spatially close to M115. Hence, methionine oxidation can disrupt (a) the loop conformation or (b) the catalytic site residue T89, among the other nonloop residues. Indeed both these treatments render the enzyme catalytically inactive, as the loop cannot attain the catalytically competent conformation. It is important to note that among the catalytic site residues, the crystal structure of the covalent intermediate of asparaginase (4ECA.pdb) also indicates the involvement of T12 in the catalysis. The microsecond dynamics broaden the T12, I13, and A14 peaks beyond detection in the methyl NMR fingerprinting of the protein but visible in the isolated peptide (Figures S19 and S20). Beyond the insights into the conformational states corresponding to catalysis (or lack of it), enzyme activity can be calculated using the apparent reaction enthalpy (Table 4). In summary, the biophysical assay allows us to profile the (a) conformational landscape of the enzyme and (b) enzymatic activity using the presence of such states.

**Table 4:** Relative Enzyme Activity of l-Asparaginase under Various Conditions, as Measured from the Apparent Enthalpy of Reaction

condition of – l-asparaginase II (in 280 μL)	solution in syringe (in 3 μL each)	no. of moles added	no. of moles converted	apparent reaction enthalpy Δ*H* (kcal/mol)	relative enzyme activity (%)
10 nM free enzyme	10 mM asparagine	0.03	8.49 × 10^–5^	–5673	100
10 nM free enzyme	10 mM (asparagine + glycine)	0.03	1.56 × 10^–5^	–1047	18
10 nM tyrosinase-treated enzyme (day 1)	10 mM asparagine	0.03	6 × 10^–5^	–4058	71.5
10 nM tyrosinase-treated enzyme (day 4)	10 mM asparagine	0.03	4.18 × 10^–5^	–2790	49.2
10 nM tyrosinase-treated enzyme (day 8)	10 mM asparagine	0.03	3.43 × 10^–5^	–2318	41

### Scope of Validity

The combined biophysical assay was also applied for l-asparaginase II in a different buffer, e.g., borate pH 8.0 to gauge the scope of validity under different buffer conditions. The reaction enthalpy is positive (+195 kcal), as shown by ITC (Figure S21A). The methyl NMR spectra of asparaginase in the presence and absence of asparagine also do not show any peak broadening for various residues, as determined before (Figure S21B). The reaction heat is closer to the asparagine dilution heat, i.e., +120 cal (Figure S22). Both these findings suggest that the reaction does not proceed in the borate buffer. The synergistic use of the biophysical methods, in this assay, correctly identifies the loss of activity of l-asparaginase under solution conditions. However, comprehensive mechanistic details of the conformational changes of asparaginase beyond the loop remain unaddressed. Since the peaks corresponding to the mobile lid loop are visible in the natural abundance spectra, the role of the other critical nonloop residues (T12, T89, D90, and K162) could not be inferred from our assay. The detailed kinetics and mechanistic insights of enzyme action remain out of scope of this work.

### Utility of Methyl Fingerprinting

Despite the limitations, the sparse methyl fingerprinting can be potentially used to probe the structure and function of proteins bearing mobile loops, i.e., **c**omplementarity-**d**etermining **r**egion loops (CDR loops) bearing monoclonal antibodies and their derivatives (Fc fusion protein and antibody drug conjugates). In a majority of these glycosylated proteins (therapeutic), labeling of amino acids cannot be performed, and hence the suite of NMR triple resonance experiments for assignment purposes cannot be performed. Such structure–function assays can also be used for rational engineering of enzymes for enhanced/altered activity. The complementary nature of NMR pulse sequences (ALSOFAST HMQC and HSQC) may allow to probe the role of different segments of the protein, of varying flexibility, in its function. Last, but not the least, such studies can be extended to other enzymes which involve segmental mobility for protein function.

## Conclusions

In this work, ITC and NMR methods have been used at natural abundance to probe the structure–function aspects of l-asparaginase. Our studies reveal the role of a dynamic lid loop of the protein that undergo conformational changes during catalytic mode. Disruption of this conformational change leads to the loss of activity of the enzyme. The above can be attained by the use of glycine or by tyrosinase/H_2_O_2_ treatment. NMR-based methyl fingerprinting of the protein allows to predict the loss of activity of the enzyme by monitoring the distinct changes in loop residues upon Asn addition. Based on the results, the disruption of loop conformation could be a sufficient but not a necessary condition for the loss of activity of the enzyme.

## Supporting Information Available

Experimental conditions; sample descriptions; spectral results of NMR analysis; spectral correlation plots along with ITC; colorimetry; assignment scheme through which peptide assignment is transferred to the protein; relevant information obtained from X-ray crystal structures, supporting the flexibility of loop residues; location of susceptible methionine in l-asparaginase; possible interaction that the loop residues may have with other parts of the protein; HPLC studies of peroxide-oxidized samples; and working model for l-asparaginase (PDF)
