# Supplementary material for: Profiling Enzyme Activity of l-Asparaginase II by NMR-Based Methyl Fingerprinting at Natural Abundance
Source: J Am Chem Soc. 2023 May 8;145(19):10826–38. doi: 10.1021/jacs.3c02154 (PMC10197121; doi:10.1021/jacs.3c02154)
Supplement: Supplementary file 1 — ja3c02154_si_001.pdf [file ja3c02154_si_001.pdf]

Supplementary Information

Profiling enzyme activity of L asparaginase II by NMR based methyl fingerprinting at natural abundance

Rachayita Nag[1,3], Srishti Joshi[2], Anurag Singh Rathore[2], Subhabrata Majumder[1,3*]

[1]Biophysics & Structural Genomics, Saha Institute of Nuclear Physics, Kolkata 700064, India

[2]Department of Chemical Engineering, Indian Institute of Technology, Hauz Khas, New Delhi, 110016, India. [3]Homi Bhabha National Institute, Anushaktinagar, Mumbai 400094, India.

[*]Corresponding author

subhabrata.majumder@saha.ac.in

List of supplementary materials

Supplemental Tables

Table S1. Deviation of the loop residues in the overlay between free asparaginase (3ECA.pdb) and its substrate-bound mimic (4ECA.pdb).

Table S2. List of experimental and Sample conditions at 40^0 C.

Table S3. The acquisition parameters for HSQC and ALSOFAST HMQC experiment on L-asparaginase samples for comparison.

Table S4: Scheme of methyl peak assignment of the loop peptides (G10 –V30) from the truncated versions (P1…P8).

Table S5: Colorimetric assay of L asparaginase.

Table S6: Proximity of Methionine residues from the critical threonine residues of wild-type L-asparaginase.

Table S7: Proximity of critical residues from the loop residues of L-asparaginase.

Table S8. Identification of non-lid and lid loop residues of L-Asparaginase

Table S9. Impact of non-loop residues in presence of Asn and Glycine as evident from differential peak broadening

Table S10: Peak intensities of Assigned peaks of the Loop

Table S11. Intensity changes of Val $30^{\gamma1\gamma2}$ and Val $30^{\gamma1\gamma*}$ peaks across the acquired [^{1}H-^{13}C] HSQC spectra

Supplemental Figures

Figure S1: Superimposition of truncated peptides accounts for loop residue assignment.

Figure S2: Impact of temperature on the flexibility of Ile 13 of the lid loop peptide.

Figure S3. Linear regression plot of [^{1}H- ^{13}C] HSQC NMR peak intensities of loop residues between different asparaginase samples show maximum dissimilarity following Asparagine (Asn) addition.

Figure S4. Impact of glycine on the lid loop residues of L-asparaginase. The superposition of [^{1}H- ^{13}C] HSQC spectra of L-asparaginase in the presence and or absence of glycine suggests a degree of spectral similarity.

Figure S5. Tyrosinase treatment leads to altered conformation of intact L-asparaginase

Figure S6. Tyrosinase treatment alters the lid loop spectra of asparaginase.

Figure S7. Treatment of 0.5 % H_20_2 prevents characteristic spectral changes of L-asparaginase upon Asn addition.

Figure S8. Monitoring hydrogen peroxide oxidation of L-asparaginase using RP-HPLC.

Figure S9. Comparability of [^{1}H-^{13}C] HSQC and [^{1}H-^{13}C] ALSOFAST HMQC spectra of L-asparaginase.

Figure S10. Comparison of sensitivity enhancement of [^{1}H-^{13}C] HSQC spectra of L-asparaginase with (A) increase in concentration and (B) increase of temperature.

Figure S11. Spectral correlation of L-asparaginase in the presence and absence of Asn as evident from [^{1}H-^{13}C] ALSOFAST HMQC experiment.

Figure S12. Importance of non-loop residues of L-asparaginase in inhibition of enzymatic activity.

Figure S13. Orthogonality of enzymatic assay based on colorimetric and calorimetric methods.

Figure S14. Different modes of spectral changes of L-asparaginase.

Figure S15. The proximity of lid loop residue Y25 with P 117 in L-asparaginase

Figure S16. Comparison of [^{1}H-^{13}C] HSQC spectra of asparaginase in presence and absence of asparagine in 40% glycerol (v/v) at 50^0C.

Figure S17. The rotameric state of Val30 is altered in presence of L-Asn.

Figure S18. A simplistic model of the role of lid-loop in enzymatic reaction of L-asparaginase.

Figure S19. [^{1}H-^{13}C] HSQC spectra of all the peptides used.

Figure S20. [^{1}H-^{13}C] HSQC spectra of all the proteins used.

Figure S21. Asparaginase catalysis may not proceed in a borate buffer.

Figure S22. ITC thermogram showing positive apparent reaction enthalpy (+120Cal) upon addition of Asn in sodium phosphate buffer, pH 7.8.

Table **S1**: Deviation of the loop residues in the overlay between free asparaginase (3ECA.pdb) and its substrate-bound mimic (4ECA.pdb)

**3ECA Residue**	**4ECA Residue**	**Deviation of Cβ in the structural overlay (Å)**
THR 12	AEI 12*	0.347
ILE 13	ILE 13	0.196
ALA 14	ALA 14	0.371
ASP 18	ASP 18	1.286
SER 19	SER 19	0.868
ALA 20	ALA 20	**0.515**
THR 21	THR 21	**0.947**
LYS 22	LYS 22	1.715
SER23	SER 23	1.774
ASN 24	ASN 24	0.942
TYR 25	TYR 25	0.425
THR 26	THR 26	**0.35**
ALA 27	*VAL 27***	**0.920**
LYS 29	LYS 29	1.812
VAL 30	VAL 30	**1.187**

* AEI = Acyl-Enzyme-Intermediate, ** Indicates mutations

Table **S2**. List of experimental and Sample conditions at 40^0 C:

**Sample**	**Excipients**	**Concentration of Enzyme**	**Buffer composition**	**Experiment**
Bionase	None	202uM Asparaginase<br>115 uM Asparaginase (45° C)	100mM Sodium-phosphate buffer, pH 7.8	[^{1}H-^{13}C] HSQC
Bionase +Asn (1:1)	None	202uM Asparaginase	100mM Sodium-phosphate buffer, pH 7.8	[^{1}H-^{13}C] HSQC
Bionase +Gly (1:1)	None	200uM Asparaginase	100mM Sodium-phosphate buffer, pH 7.8	[^{1}H-^{13}C] HSQC
Bionase +Gly (1:1)+Asn (1:1:1)	None	200uM Asparaginase	100mM Sodium-phosphate buffer, pH 7.8	[^{1}H-^{13}C] HSQC
Modification by Tyrosinase	None	Asparaginase : Tyrosinase = 115uM – 57.5uM	50mM Potassium Phosphate buffer pH 6.5	[^{1}H-^{13}C] HSQC
H_2O_2 treated Bionase	None	1. 0.1% H2O2 treated enzyme - 91uM<br>2. 0.5% H2O2 treated enzyme - 89uM	100mM Sodium Phosphate buffer pH 7.8	[^{1}H-^{13}C] HSQC
Bionase + D-Aspartate (1:1)	None	207uM Asparaginase	100mM Sodium Phosphate buffer pH 7.8	[^{1}H-^{13}C] HSQC
Bionase + L-Aspartate (1:1)	None	140uM Asparaginase	100mM Sodium Phosphate buffer pH 7.8	[^{1}H-^{13}C] HSQC
Bionase +Asn (1:1) in BB	None	115uM	50mM Borate buffer, pH 8.0	[^{1}H-^{13}C] HSQC
Bionase	Yes	150 µM	Water	1. [^{1}H-^{13}C] HSQC<br>2. [^{1}H-^{13}C] ALSOFAST HMQC
Bionase +Gly (1:1)	No	150 µM	100mM Sodium Phosphate buffer, pH 7.8	1. [^{1}H-^{13}C] HSQC<br>2. [^{1}H-^{13}C] ALSOFAST HMQC
Bionase +Gly + Asn (1:1:1)	No	150 µM	100mM Sodium Phosphate buffer, pH 7.8	[^{1}H-^{13}C] ALSOFAST HMQC

Table S3. The acquisition parameters for HSQC and ALSOFAST HMQC experiment on L-asparaginase samples for comparison :

**Samples**	**Experiment**	**Protein concentration (uM)**	**Tempe rature (□C)**	**Scan**	**Acquisition time (Sec) (f2-f1)**	**Spectral Width (Ppm) (f2-f1)**	**FID POINT (Hz)**	**D1 (Sec)**
Asparaginase in water	hsqcetgpsi	150	40	160	0.0655 - 0.0189	19.5329-29.8000	15.258-53.045	1.20
Asparaginase in water	afhmqcgpphsf	150	40	160	0.0655-0.0189	19.5329-29.8000	15.258-53.045	0.40
Asparaginase in sodium phosphate buffer, pH 7.8	hsqcetgpsi	156	40	160	0.0655-0.0189	19.5329-29.8000	15.258-53.045	1.20
Asparaginase in sodium phosphate buffer, pH 7.8	afhmqcgpphsf	156	40	160	0.0655-0.0189	19.5329-29.8000	15.258-53.045	0.40
Asparaginase, Glycine, and Asparagine in sodium phosphate buffer, pH 7.8	afhmqcgpphsf	150	40	160	0.0655-0.0189	19.5329-29.8000	15.258-53.045	0.40

Table **S4**: Scheme of methyl peak assignment of the loop peptides (G10 –V30) from the truncated versions (P1,…P8)

	Intact loop peptide	P 1	P 2	P 3	P 4	P5	P 6	P 7	P 8
Intact loop peptide	-								
P 1	**T12**	**-**							
P 2		**Ile13**	**-**	**A20****					
P 3			**A27***	**-**					
P 4			**T26**		**-**				
P 5					**V30**	**-**			
P 6						**T21**	**-**		
P 7								**-**	
P 8								**A14 *****	**-**

*Assigned by elimination, **Peak overlapping in intact peptide, Table ***Broadened out in the intact protein much like I13 and T12

Table **S5**: **Colorimetric assay of L asparaginase**

	Condition (1)			Condition (2)	
Reactants$^{\gamma}$	Blank (µL)	Bionase (µL)	Reactants	Blank (µL)	Bionase (µL)
Nessler's (162µM)	200	200	Nessler's (1mM)	50	50
50mM Tris (pH 8.6)	149	147	50mM Tris (pH 8.6)	300	100
Water	146	146	Water	100	100
Substrate (**107µM**)	5.4	5.4	Substrate (**1mM**)	50	50
Enzyme (**10nM**)	-	2ul	Enzyme **(1µM)**	-	200

γ reaction volume = 500 µL.

Table **S6:** Proximity of Methionine residues from the critical threonine residues of wild-type L-asparaginase.

Methionine Residues spatially close to the loop	Distances between atoms in 3ECA.pdb					
	T12 (Loop)		T21 (Loop)		T89 (Non-loop residue)	
Met 115	6.71(SD-CG2)	7.02 (CG-CG2)	-		3.73 (SD-CG2)	4.34 (CG-CG2)
Met 121	-		3.04 (SD-CG2)	3.87 (CG-CG2)	-	

Table **S7**: Proximity of critical residues from the loop residues of L-asparaginase

		Distances with loop residues of 3ECA.pdb (Asp –NH to loop residue-Cβ in Å)				Distances with loop residues of 4ECA.pdb			
		T21	T26	A27	V30	T21	T26	A27	V30
A.	Product Aspartate	19.74	9.00	4.32	13.75	-			
		Y25 (Cβ- Cβ in Å)				Y25 (Cβ- Cβ in Å)			
B.	Proline 117	4.01				3.70			

Table **S8**. Identification of non lid and lid loop residues of L-Asparaginase

**Peak Numbers**	**Protein HSQC**	**Loop peptide (HSQC)**	**ALSOFAST HMQC**
1**a.** Methyl peaks, part of the flexible loop	√	√	√
**1b**. Methyl peaks, part the non-loop region	√	×	√
**1c**[α]. Non-methyl loop peptide peak	√	√	√
**2a**[α]**.** Non-methyl part of the flexible loop	√	√	×
**2b**. Non-methyl, non-loop region	√	×	×

[α] may correspond to Lysine methylene groups of the loop (K22, K29)

Table S9. Impact of non-loop residues in presence of Asn and Glycine as evident from differential peak broadening*.

Peaks	free	(+Asn)	(+gly)	(gly,Asn)
a	□□	□□	√	□□
b	√	□	□	□
c	□	√*	√	□□
d	√	√	√	□□
e	√	√	√	□□
f	□	√	√	√
g	√	□	□□	□□
h	√	□	□□	√

The extent of broadening □□> □. Unaffected = √

Table **S10**: Peak intensities of Assigned peaks of the Loop:

Peaks	Val 30$^{\gamma1\gamma2}$*	Ala 27	Ala 20	Val$^{\gamma1\gamma2}$	Thr 26	Thr 21
**S1**	9.24E+08	1.36E+09	1.92E+09	1.36E+09	1.95E+09	1.14E+09
**S2**	4.51E+08	6.67E+08	1.24E+09	9.19E+08	8E+08	5.74E+08
**S3**	3.7E+08	6.71E+08	8.55E+08	7.78E+08	8.89E+08	6.54E+08
**S4**	4.76E+08	4.81E+08	1.05E+09	8.58E+08	6.32E+08	7.65E+08
**S5**	1.52E+08	3.85E+08	3.69E+08	2.49E+08	4.9E+08	7.19E+08
**S6**	1.68E+08	3.7E+08	7.39E+08	2.43E+08	4.88E+08	5.5E+08
**S7**	4.66E+08	6.38E+08	7.72E+08	6.81E+08	8.31E+08	3.41E+08
**S8**	4.32E+08	7.4E+08	7.96E+08	7.76E+08	7.88E+08	8.09E+08
**S9**	68203032	1.08E+08	1.26E+08	96664640	1.29E+08	1.26E+08
**S10**	9.24E+08	1.36E+09	1.92E+09	1.36E+09	1.95E+09	1.14E+09
**S11**	9.78E+08	1.28E+09	1.6E+09	1.14E+09	1.63E+09	1.29E+09
**S12**	1.27E+08	2.99E+08	2.6E+08	5.38E+08	4.47E+08	2.58E+08

Experiment details :

S1 =Asparaginase Peak intensities, S2 =Asparaginase + Asn Peak intensities, S3 =Glycine added asparaginase Peak intensities, S4 =Glycine added asparaginase + Asn Peak intensities, S5 =Tyrosinase treated asparaginase Peak intensities, S6 = Tyrosinase treated asparaginase + Asn Peak intensities, S7 =0.1% H_2O_2 treated asparaginase Peak intensities, S8 =0.5% H_2O_2 treated asparaginase Peak intensities, S9 =0.5% H_2O_2 treated asparaginase + Asn Peak intensities, S10 =Asparaginase + L- aspartate Peak intensities, S11 =Asparaginase + D-aspartate Peak intensities, S12 =36% Glycerol added Asparaginase.

Table **S11.** Intensity changes of Val $30^{\gamma1\gamma2}$ and Val $30^{\gamma1\gamma^{*}}$ peaks across the acquired [^{1}H-^{13}C] HSQC spectra.

**Sample description**	**Free (Val $30^{\gamma1\gamma2}$/Val $30^{\gamma1\gamma2*}$) peak intensity ratio (X)**	**Substrate added (Val $30^{\gamma1\gamma2}$/Val $30^{\gamma1\gamma2*}$) peak intensity ratio (Y)**	**The difference in the peak intensity ratio (Y-X=Z)**	**Fold change (Z/X*100)**	**Reaction heat obtained from ITC at 25°C (in kcal/mol)**
Asparaginase at 40°C	1.47	2.04	0.57	37%	-5.6
Asparaginase at 45°C	1.12	1.78	0.66	59%	-5.6
Asparaginase + Glycine (1:1) at 40°C	1.76	1.47	-0.29	-16.4%	-1.04
Asparaginase + Tyrosinase (2:1) at 40°C	1.48	1.62	0.14	9.5%	-2.8
0.5% Methionine-treated Asparaginase, at 40°C	1.79	1.47	-0.32	-17.9%	NA
Asparaginase + L-Asp at 40°C	1.47	1.19	-0.28	-19%	NA
Asparaginase + D-Asp (45°C)	1.12	1.32	-0.20	-17.8%	NA

Supplementary Figures

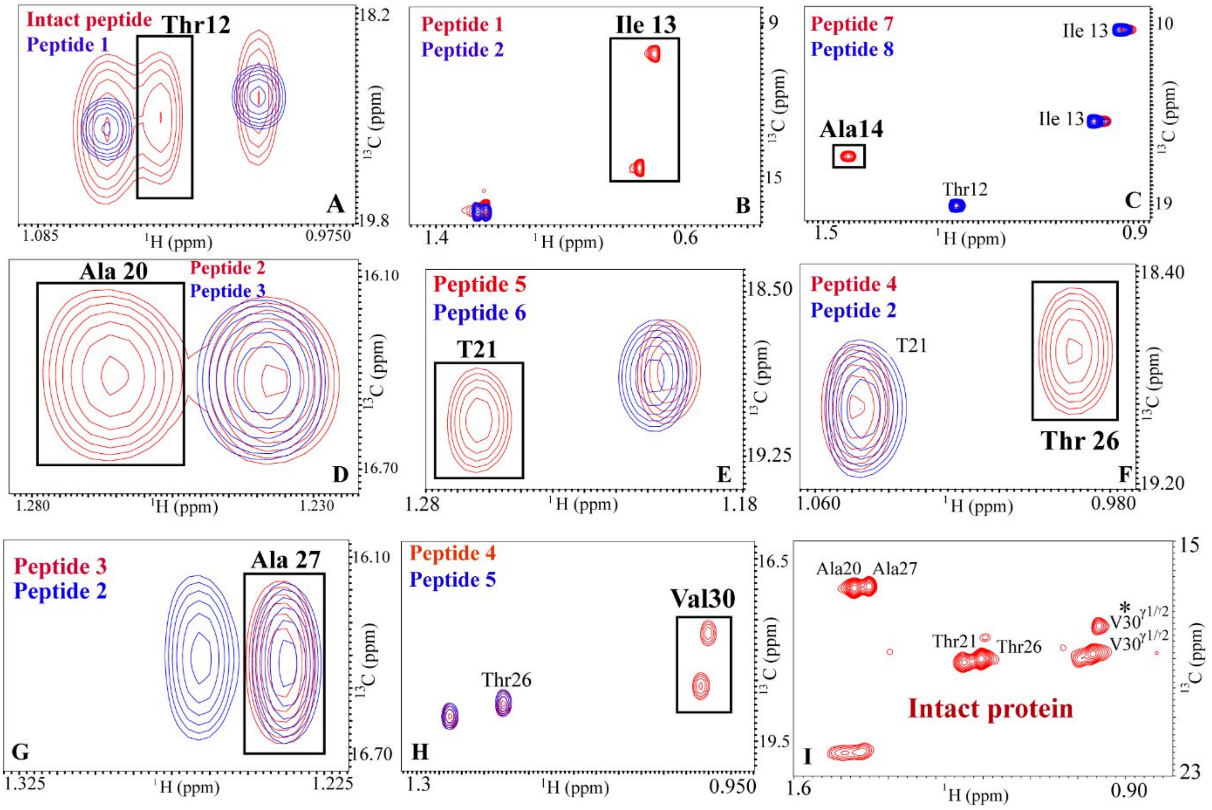

Figure S1: Superimposition of truncated peptides accounts for loop residue assignment. A) Superimposing the intact peptide spectra with that of peptide 1, assigns residue **T12**. B) Superposition of peptide 1 and peptide 2 allowed for the assignment of **I13**. C) Superimposition of peptide 7 with peptide 8, enabled to assign **A14**. D) Assignment of **A20** was done by the superimposition of the spectra of peptides 2 and 3. E) Superposition of peptide 5 and peptide 6 leads to the assignment of **T21**. F) Similarly assignment of **T26** was obtained from the superposition of the spectra of peptide 2 and peptide 4. G) Assignment of **A27** was obtained from the superposition of peptide 7 and peptide 8. H) Assignment of **V30** was obtained by comparing the spectra of peptide 4 and peptide 5. I) Assignment of the residues transferred to the intact protein.

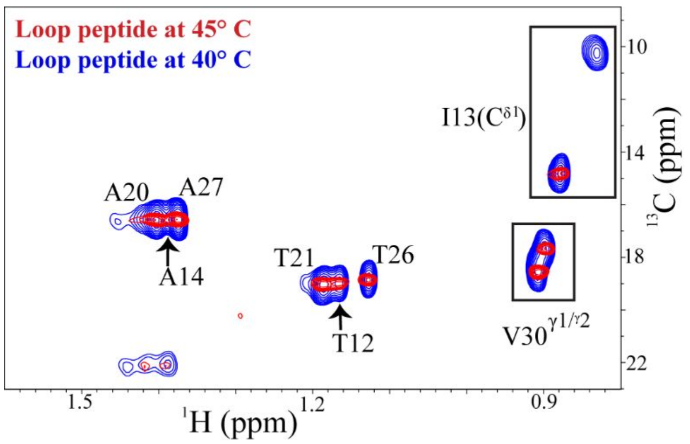

Figure S2: **Impact of temperature on the flexibility of Ile 13 of the lid loop peptide.** The spectra of the chemically synthesized loop show a differential broadening of I13 peaks in [^{1}H-^{13}C] HSQC spectra at 45^{0} C, compared to that of 40^{0} C, suggesting increased flexibility of the side chains. At higher temperatures (45^{0} C) one of the rotameric states corresponding to I13 $^{\delta}$C gets broadened out.

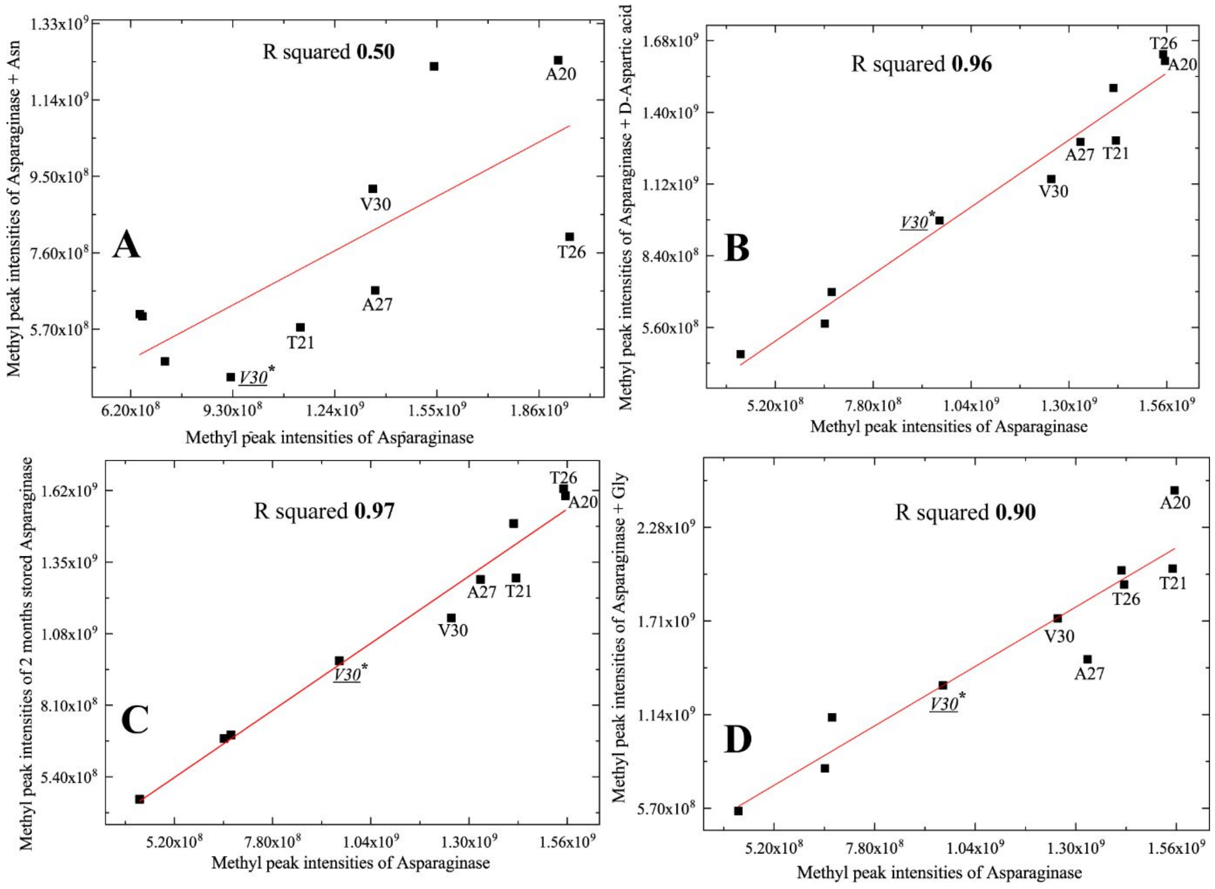

Figure S3. Linear regression plot of [^{1}H- ^{13}C] HSQC NMR peak intensities of loop residues between different asparaginase samples show maximum dissimilarity following Asparagine (Asn) addition. (A) The addition of Asn to asparaginase resulted in significant dissimilarity (R^2 value = 0.5) in methyl peak intensities when plotted against free asparaginase. Most of the loop residues appear as distinct outliers suggesting specific broadening at those residues. (**B)** The regression plot of methyl peak intensities of D-Aspartic acid added asparaginase with free enzyme showed no significant dissimilarities (R^2 value = 0.96) in peak intensities between the two. **(C)** A linear regression plot of methyl peak intensities between asparaginase duplicate samples indicated both spectra as nearly identical (R^2 value = 0.97). **(D)** The addition of Gly to asparaginase resulted in no significant dissimilarities (R^2 value = 0.90) in methyl peak intensities when plotted against free asparaginase.

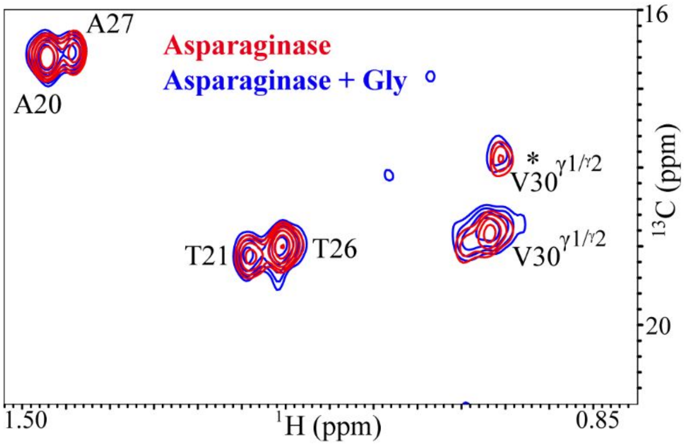

Figure S4. Impact of glycine on the lid loop residues of L-asparaginase. The superposition of [^{1}H- ^{13}C] HSQC spectra of L-asparaginase in the presence and or absence of glycine suggests a high degree of spectral similarity as evident from the correlation coefficient 0.9.

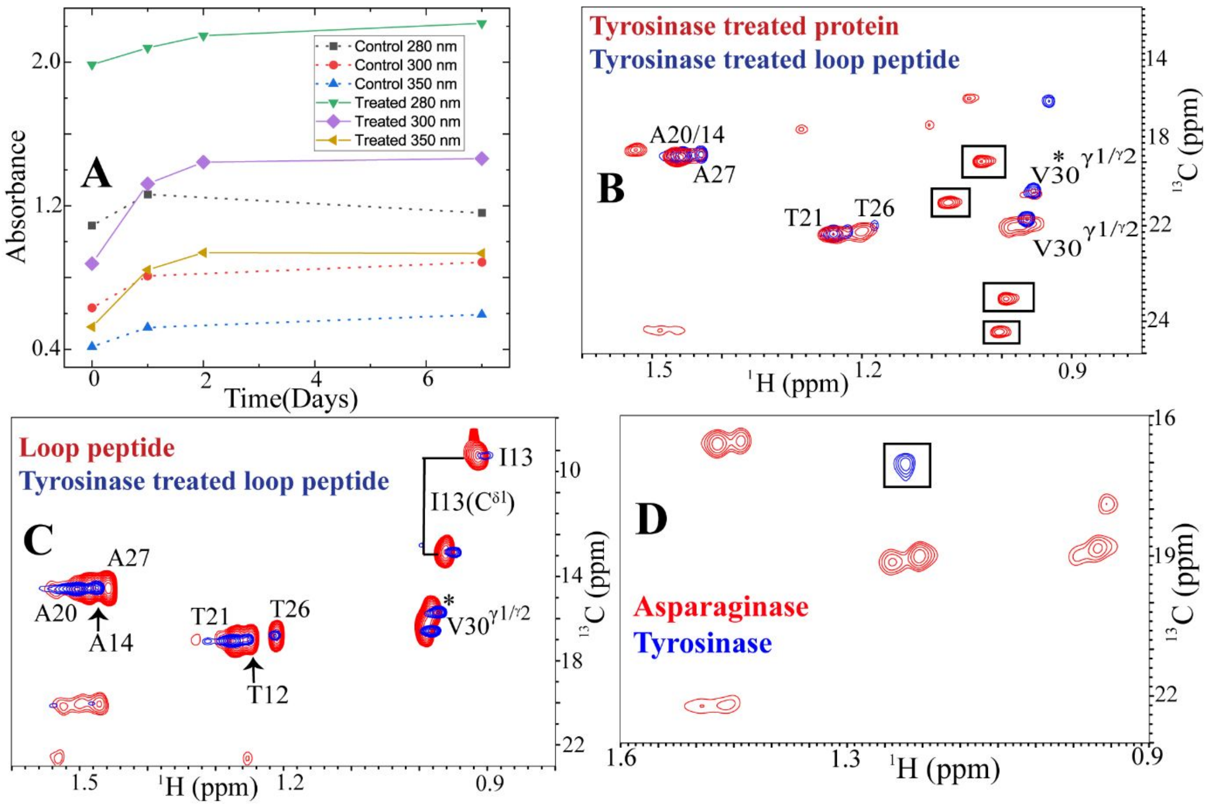

Figure S5. Tyrosinase treatment leads to altered conformation of intact L-asparaginase. **(A)** The graphical representation shows the increase in absorbance value (at 280nm, 300nm, 350nm) of tyrosinase-treated asparaginase over time. The control enzyme's (untreated asparaginase) absorbance doesn't increase significantly & remains unaltered over time. **(B)** [^{1}H- ^{13}C] HSQC HSQC spectral overlay of Tyrosinase treated protein (in Red) with Tyrosinase treated loop peptide (in Blue) showed the appearance of new peaks in the treated protein. The new peaks are marked with a rectangle. **(C)** 2D [^{1}H- ^{13}C] HSQC spectral overlay of untreated loop peptide (in Red) with Tyrosinase treated loop peptide (in Blue) didn't show the appearance of any new peaks, unlike that in the treated protein. **(D)** 2D methyl spectral overlay of 2D [^{1}H-^{13}C-] HSQC spectra of asparaginase (+tyrosinase) (in Red) with enzyme Tyrosinase (in blue) alone demonstrated only a single methyl peak signature by Tyrosinase (marked with a rectangle). This peak didn't match with the newly appeared peaks found in the tyrosinase-treated asparaginase. Hence, the possibility of the new peaks coming from tyrosinase itself got eliminated.

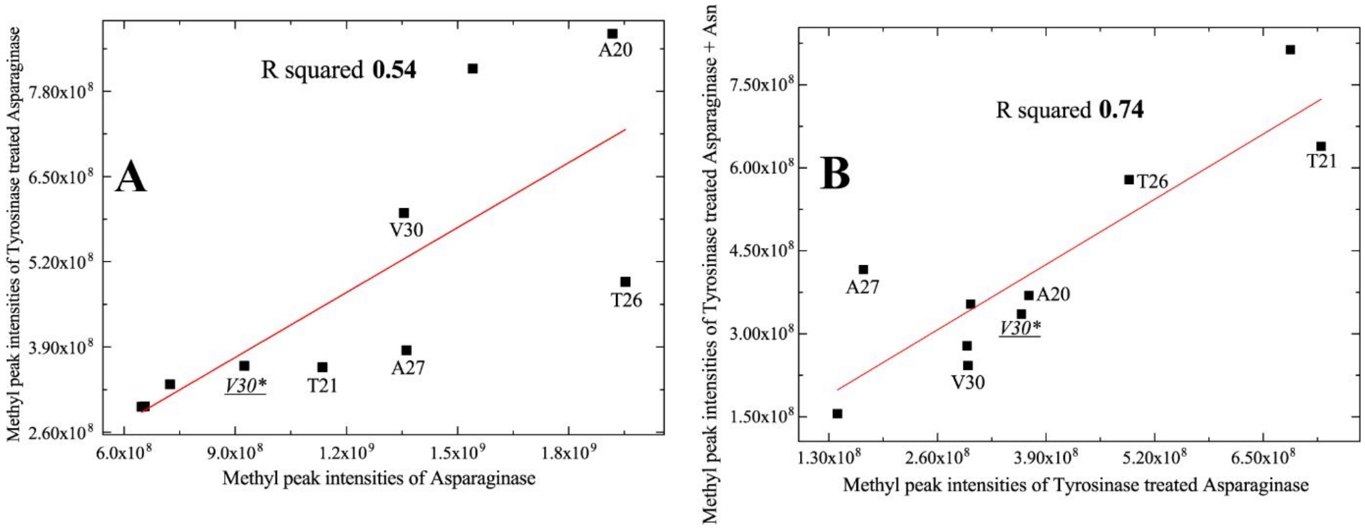

Figure S6. Tyrosinase treatment alters the lid loop spectra of asparaginase. Comparison of methyl peak intensities of [^{1}H- ^{13}C] HSQC spectra of tyrosinase-treated asparaginase and control protein shows linear regression coefficient R^2 =0.54. The same between tyrosinase-treated protein in the presence and absence of Asn shows a linear regression coefficient of 0.74.

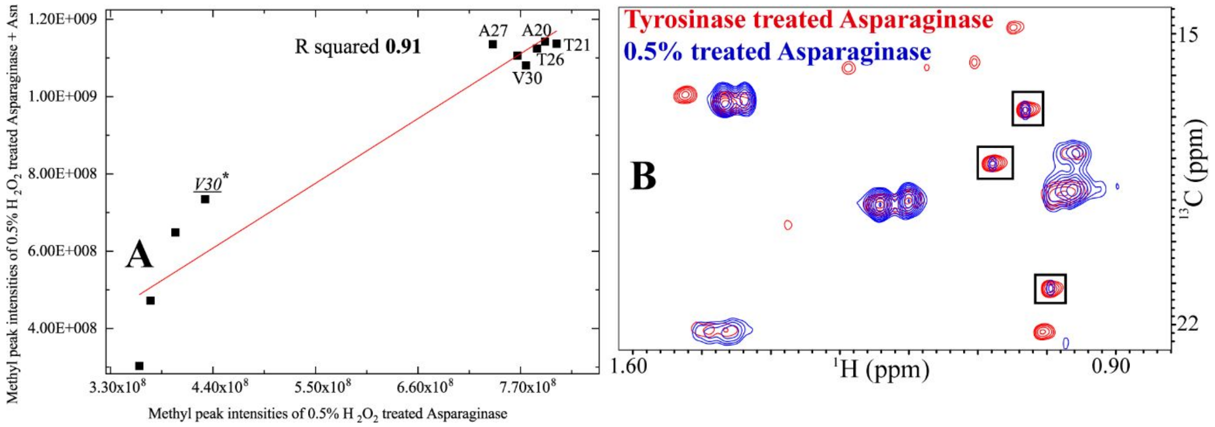

Figure S7. Treatment of 0.5 % H_20_2 prevents characteristic spectral changes of L-asparaginase upon Asn addition. (A) Overlay of 2D [^{1}H-^{13}C] HSQC spectra of asparaginase, treated with 0.5 % H_20_2, in the presence and absence of Asn is nearly identical (R^2 = 0.91). This suggests the presence of Asn does not alter the lid loop conformation of 0.5 % H_20_2 treated Asparaginase. **(B)** The new peaks in 2D [^{1}H-^{13}C] HSQC spectra of asparaginase, treated with 0.5 % $H_20_{2,}$ overlay with a subset of peaks in tyrosinase-treated L asparaginase (marked by the rectangles). This shows both tyrosinase and 0.5 % H_20_2 treatment, induce a similar conformational change in L asparaginase, which may or may not be limited to the lid loop residues.

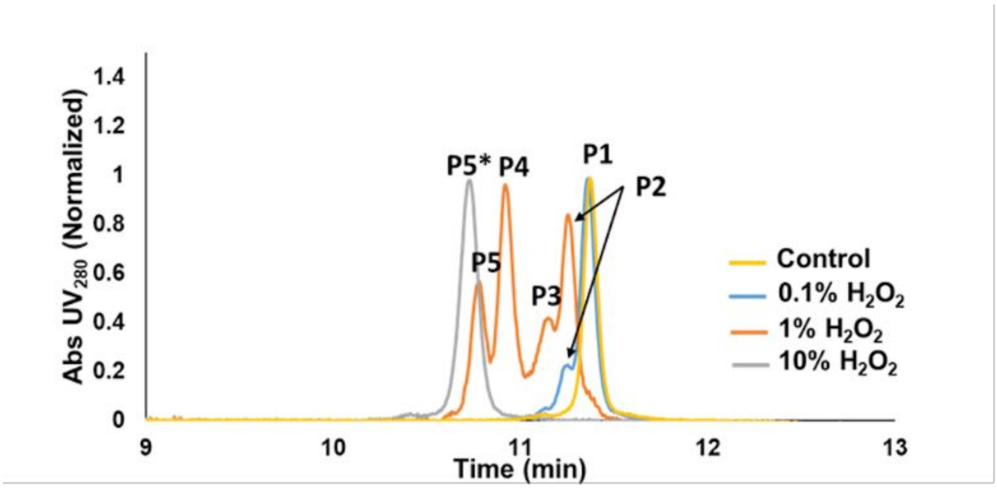

Figure S8. Monitoring hydrogen peroxide oxidation of L-asparaginase using RP-HPLC. Incubation of L-Asparaginase with increasing H_2O_2 (%), namely 0 % (control, yellow), 0.1% (blue), 1% (orange), and 10% (grey) H_2O_2 (v/v) for 14 h led to the generation of variants with increasingly reduced hydrophobicity as resolved chromatographically using reverse phase separation (representative chromatogram). P1-P5 denotes distinct chromatographic peaks resolved. Overlapping but significantly ($P<0.05$) distinct peaks are indicated with an * following the numeral.

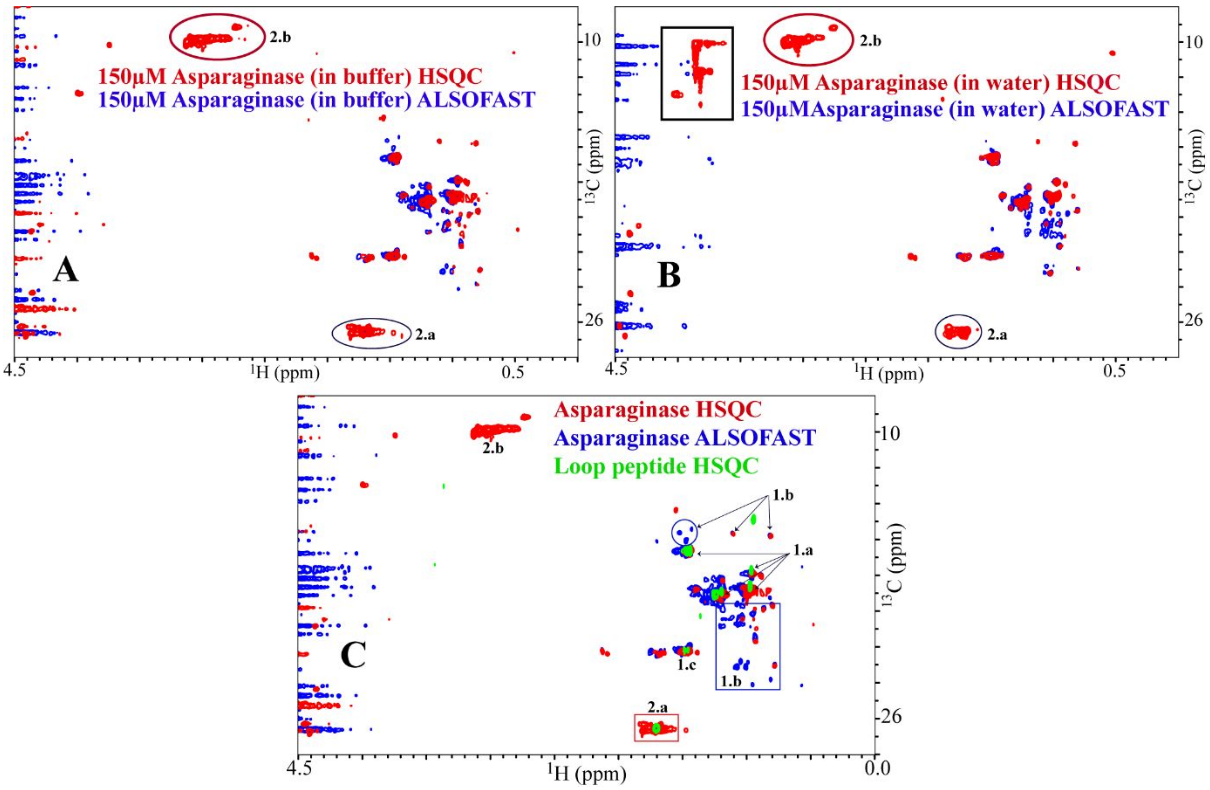

Figure S9. Comparability of [^{1}H-^{13}C] HSQC and [^{1}H-^{13}C] ALSOFAST HMQC spectra of L-asparaginase. (A) Overlay of [^{1}H-^{13}C] HSQC and [^{1}H-^{13}C] ALSOFAST HMQC spectra of buffer dialyzed identical sample of L-asparaginase (150 μM) at 40^0 C. Although the acquisition time for the ALSOFAST experiment is less (~5 hours), there is a sensitivity gain compared to the [^{1}H-^{13}C] HSQC experiment (~13 hours). This is evident from the increased number of peaks in ALSOFAST spectra compared to HSQC spectra. **(B)** Such an effect is also evident in a water reconstituted sample of L-asparaginase, which may have interfering excipient signals (in the black square). **(C)** Comparison of [^{1}H-^{13}C] HSQC and [^{1}H-^{13}C] ALSOFAST HMQC spectra of L-asparaginase and [^{1}H-^{13}C] HSQC of the loop peptide shows that peaks corresponding to the lid loop of L-asparaginase have no additional sensitivity increase in ALSOFAST HMQC experiment. However, for the non-lid loop residues, there is a distinct sensitivity enhancement in the ALSOFAST HMQC spectra of L-asparaginase. These additional peaks, with no corresponding peaks in the lid loop peptide, have lower intensities, unlike the flexible methyl-bearing lid loop residues. Hence it is possible that ALSOFAST HMQC offers a sensitivity gain for structured/less mobile non-lid loop methyl-bearing residues. The peaks are labeled and categorized as shown in Table S**8**.

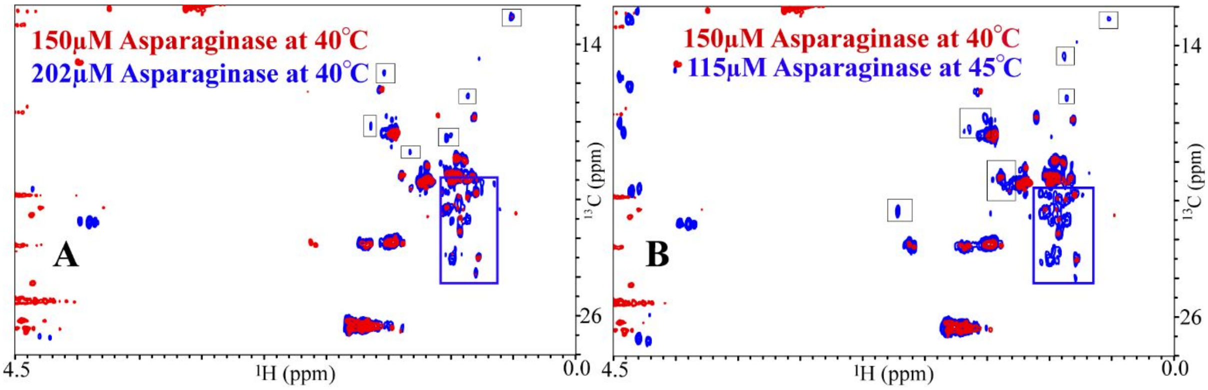

Figure S10. Comparison of sensitivity enhancement of [^{1}H-^{13}C] HSQC spectra of L-asparaginase with (A) increase in concentration and **(B)** increase of temperature. In both cases, spectral sensitivity is enhanced as evident from the appearance of peaks of non-lid loop residues. The increase in sensitivity of HSQC with an increase in temperature is similar to that of the ALSOFAST HMQC spectra. The blue box denotes additional peaks at high temp/high concentrations.

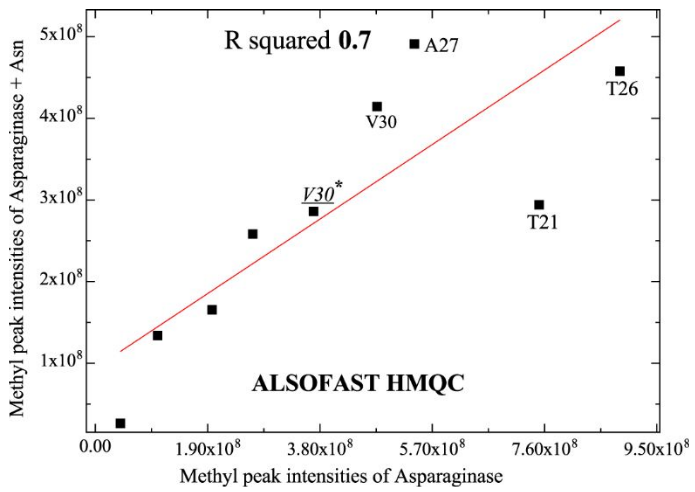

Figure S11. Spectral correlation of L-asparaginase in the presence and absence of Asn as evident from [^{1}H-^{13}C] ALSOFAST HMQC experiment. The correlation coefficient is higher in ALSOFAST HMQC (R^2 = 0.7), compared to the methyl HSQC experiment (R^2 =0.5, Table 2), using the same set of peaks. Thus HSQC is more sensitive to differential peak broadening of lid loop residues upon Asn addition.

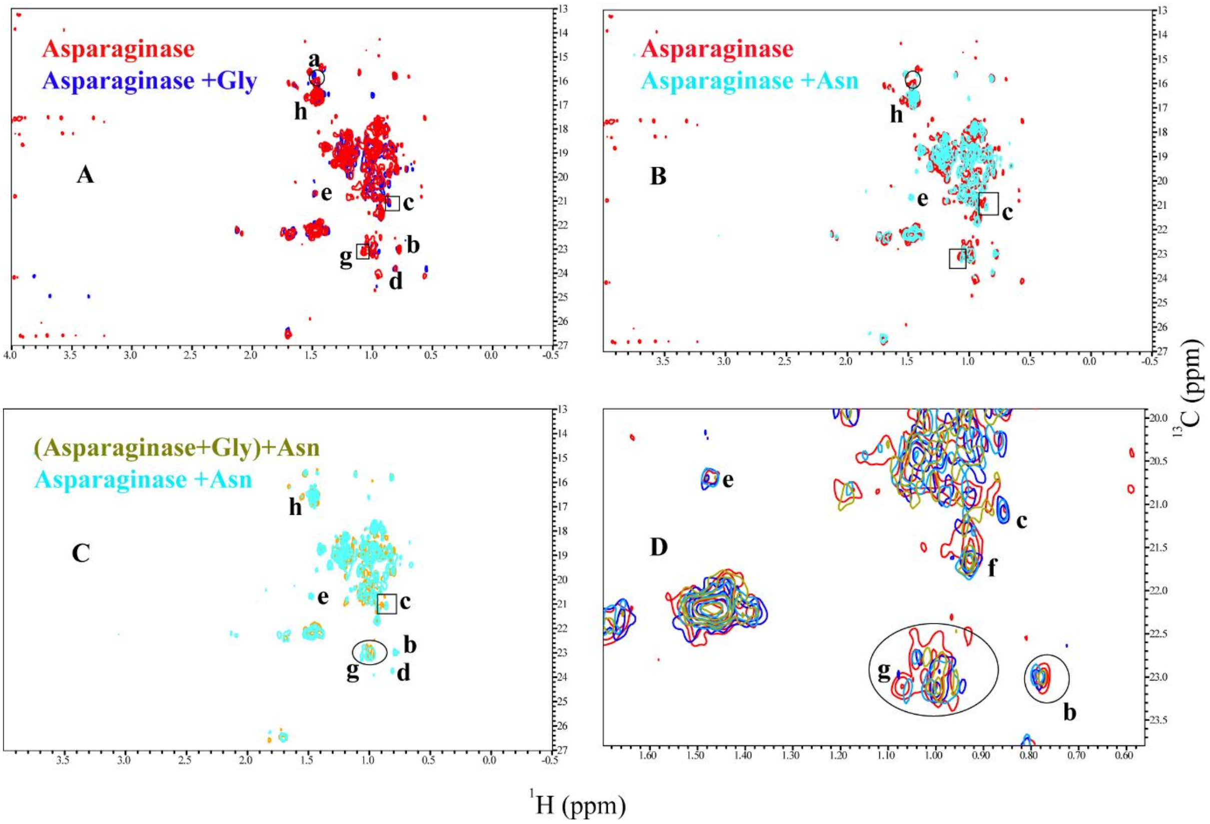

Figure S12. Importance of non-lid loop residues of L-asparaginase in inhibition of enzymatic activity. (A) Overlay of [^{1}H-^{13}C] ALSOFAST HMQC of L-asparaginase (150 µM) at 40^{0} C in the presence and absence of glycine shows differential peak broadening of peak **c,g,** and appearance of peak **a** upon glycine addition. (B) Overlay of [^{1}H-^{13}C] ALSOFAST HMQC of L-asparaginase (150 µM) in the presence and absence of Asn shows differential peak broadening of peak **c,g.** (B) Overlay of [^{1}H-^{13}C] ALSOFAST HMQC of L-asparaginase (150 µM) in presence of (Gly+ Asn) and asn only shows distinct differences in peak intensities. For example, peak **e** is differentially broadened for Asn only. Similarly, the peak intensity of **c** is unchanged in presence of (Gly+Asn). (D) Overlay of the cross-section of [^{1}H-^{13}C] ALSOFAST HMQC of L-asparaginase (150 µM) in presence of the above conditions suggests a differential broadening of peak g,f in presence of all the ligands. Thus methyl fingerprinting of non-loop residues can be used to identify the conformational diversity of the protein under different conditions. Specifically, Asn mediated dynamics of L asparaginase is unique and cannot be reconstituted in presence of Gly or (Gly+Asn). Functionally, Asn hydrolysis by asparaginase is inhibited in presence of an equimolar amount of Glycine. The differentially broadened peaks (discussed above) in all cases are shown in Table S9.

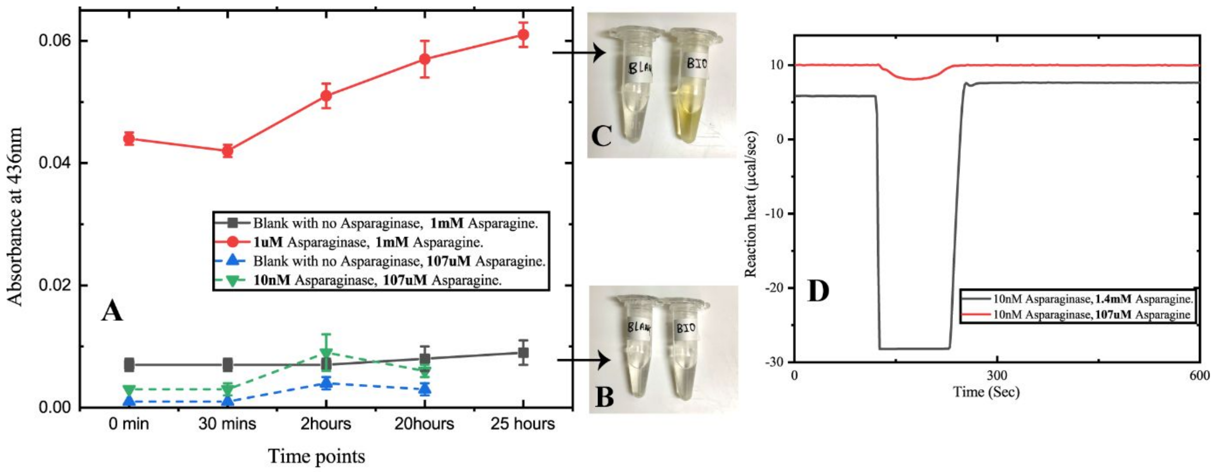

Figure S13. Orthogonality of enzymatic assay based on colorimetric and calorimetric methods. (A) Nessler's assay-based method was used to detect the activity of L-asparaginase, using the absorbance at 436 nm, where the color change is due to the complexation of ammonia (resulting from hydrolysis of Asn) and Nessler's reagent. The change in coloration appears to be strongly dependent on substrate Asn concentration. As long as the Asn concentration was around 107 µM (ITC relevant), no change in absorbance was detected with 10 nM Asparaginase or with 1 µM Asparaginase (data not shown). The absorbance value stayed close to that of the blank. **(B)** Correspondingly there was no visual color difference between the blank and enzyme-substrate sample **(C)** Distinct change in absorbance was noted when substrate concentration was changed to 1mM. **(D)** On the other hand, there is discernable heat (~-5.4kcal/mole) in ITC-relevant conditions. Increasing substrate concentration to mM range saturates the detector which renders the method unusable. Thus, while ITC is more sensitive to detecting asparaginase activity at lower concentrations, colorimetric assays are more suitable in higher substrate concentrations.

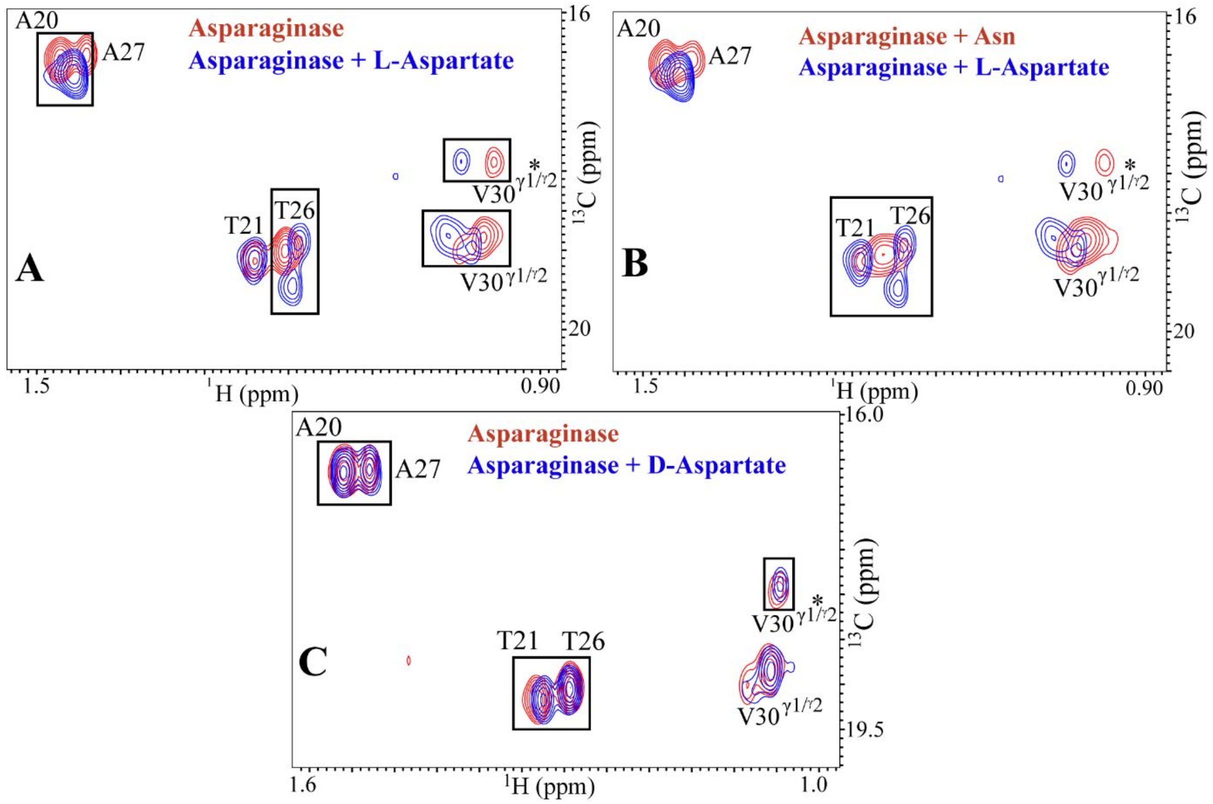

Figure S14. Different modes of spectral changes of L-asparaginase. Overlay of 2D [^{1}H-^{13}C-] HSQC spectra of **(A)** Asparaginase in the presence and absence of L-aspartate, **(B)** Asparaginase + L-Asn and Asparaginase + L aspartate, **(C)** Asparaginase in the presence and absence of D-aspartate. Hence, L-Asn addition induces a very specific change in L-Asparaginase spectra which corresponds to the catalytic mode. This is different from L-aspartate binding to the loop or from D-aspartate addition (non-binder).

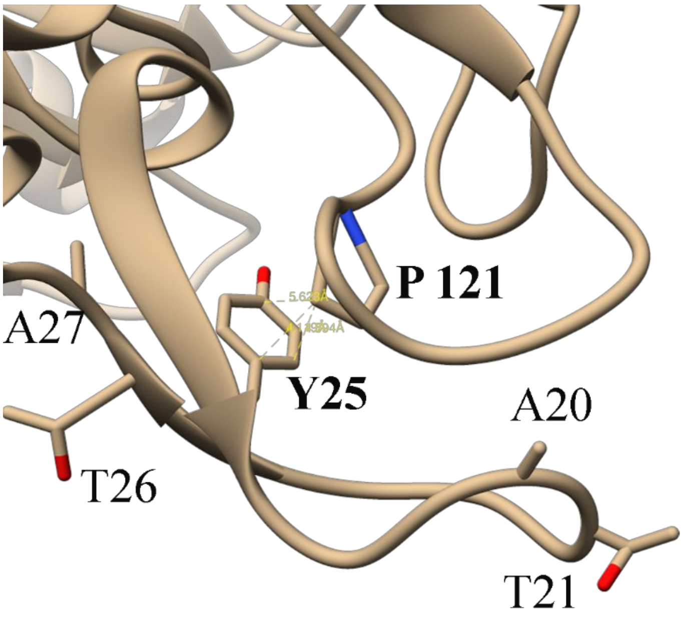

Figure S15. The proximity of lid loop residue Y25 with P 117 in L-asparaginase. The addition of L-Asn or L-Asp may alter such interaction, potentially impacting residue T26 of the loop.

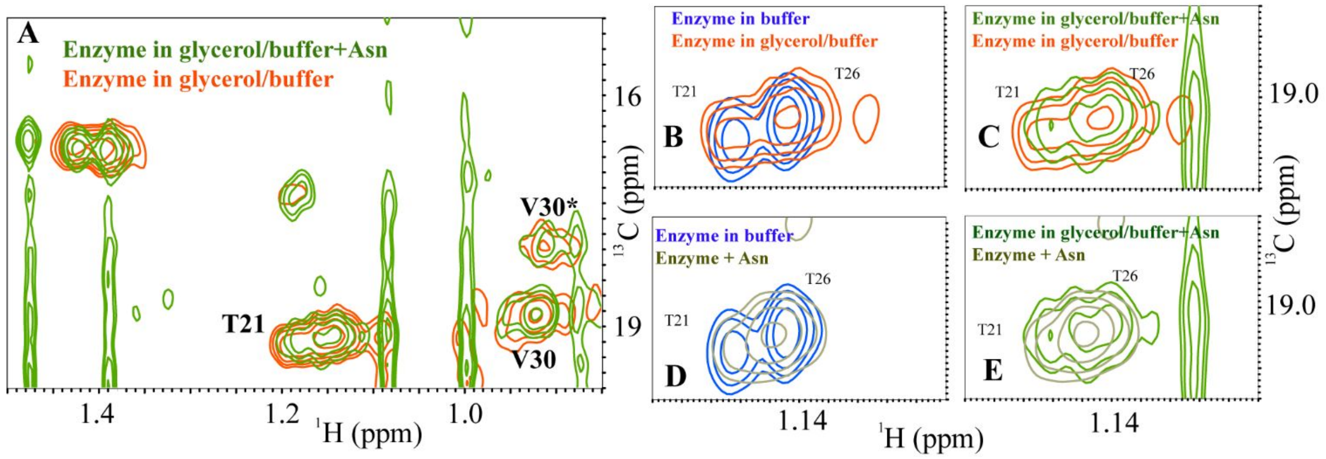

Figure S16: Comparison of [^{1}H-^{13}C] HSQC spectra of asparaginase in the presence and absence of asparagine in 40% glycerol (v/v) at 50^{0}C. (A, B) The peaks corresponding to lid loop residues (T21, T26) display inhomogeneous peak broadening. This may indicate the presence of alternate protein (lid loop) conformations in slow exchange time scales in glycerol, otherwise absent in buffer. **(C, D)** Addition of Asn causes inhomogeneous peak broadening in glycerol buffer as well but is not identical to that in the buffer. It is possible that glycerol may stabilize intermediate conformations of the lid loop in both in presence and absence of Asn otherwise absent in the buffer. These conformation(s) may exist in slow exchange time scales

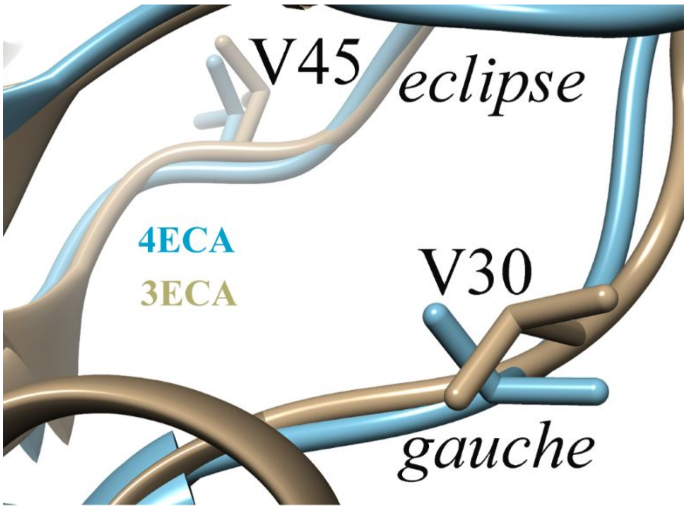

Figure S17. The rotameric state of Val30 is altered in presence of L-Asn. Superimposition of 3ECA.pdb (grey) and 4ECA.pdb (turquoise) by using UCSF Chimera software. Bond-Atoms representations of Val residues showed the presence of Gauche state, particularly, in Val30 residue, whereas, the other Val (only Val45 is shown in the figure) prevails in eclipse conformation.

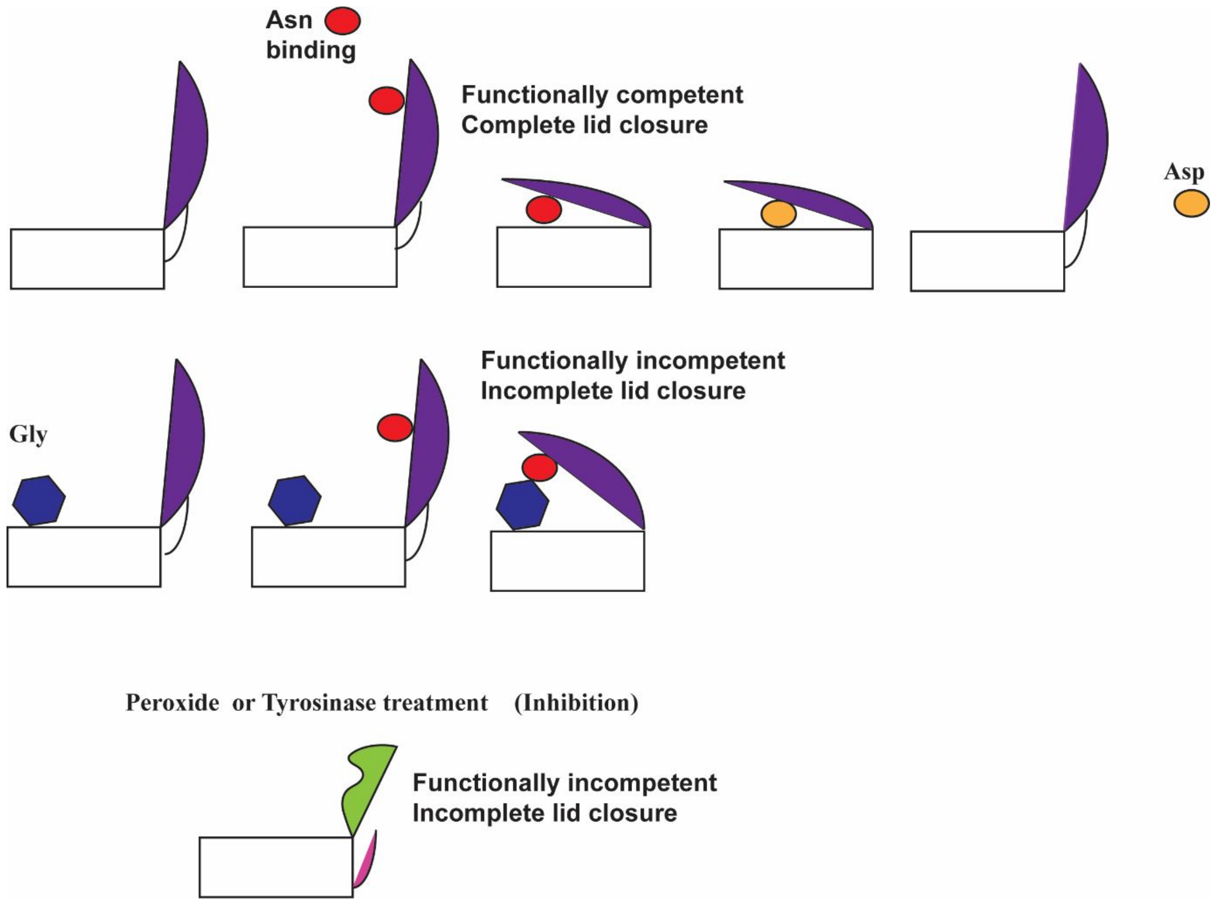

Figure S18. A simplistic model of the role of lid-loop in enzymatic reaction of L-asparaginase. Colored segments are NMR visible (lid loop) whereas white regions are not NMR visible using the natural abundance sample. In presence of Asn, the lid loop undergoes a conformational change, which is manifested by the broadening of residues T21, T26, A27, and V30 as evident from [^{1}H- ^{13}C] HSQC spectra of Asparaginase in presence of Asn. In presence of Glycine, there is no conformational change in the lid loop as evident from [^{1}H- ^{13}C] HSQC spectra. However, ALSOFAST HMQC suggests that the structured part of the protein may be involved in interaction with glycine (white box). This prevents the normal lid loop closure when Asn is added preventing the catalysis to happen. Hydrogen peroxide-mediated oxidation alters the loop conformation of the lid loop and/or the protein in general due to the oxidation of methionine (inferred from HPLC). This results in a loss of activity.

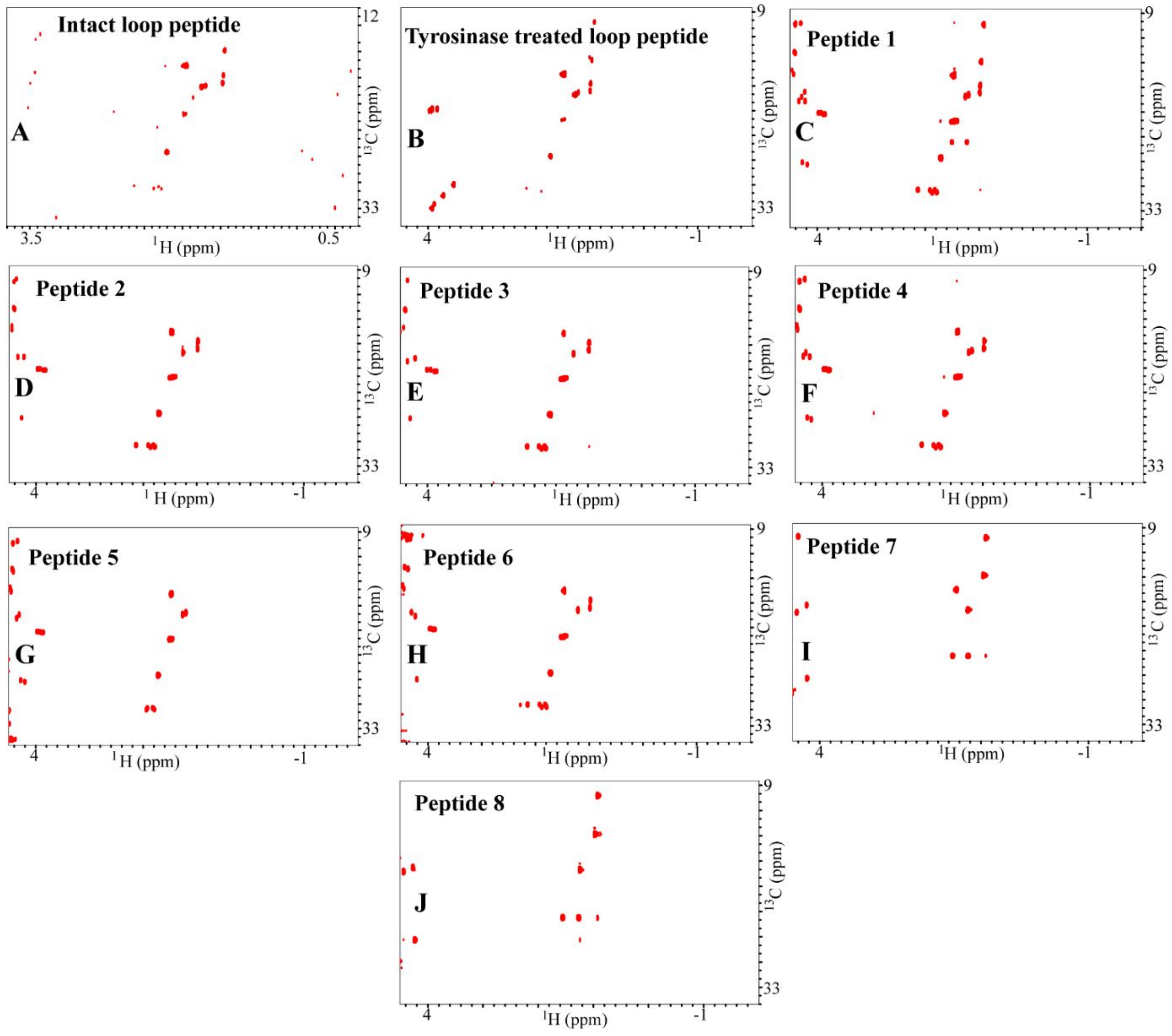

Figure S19. [^{1}H-^{13}C] HSQC spectra of all the peptides used.

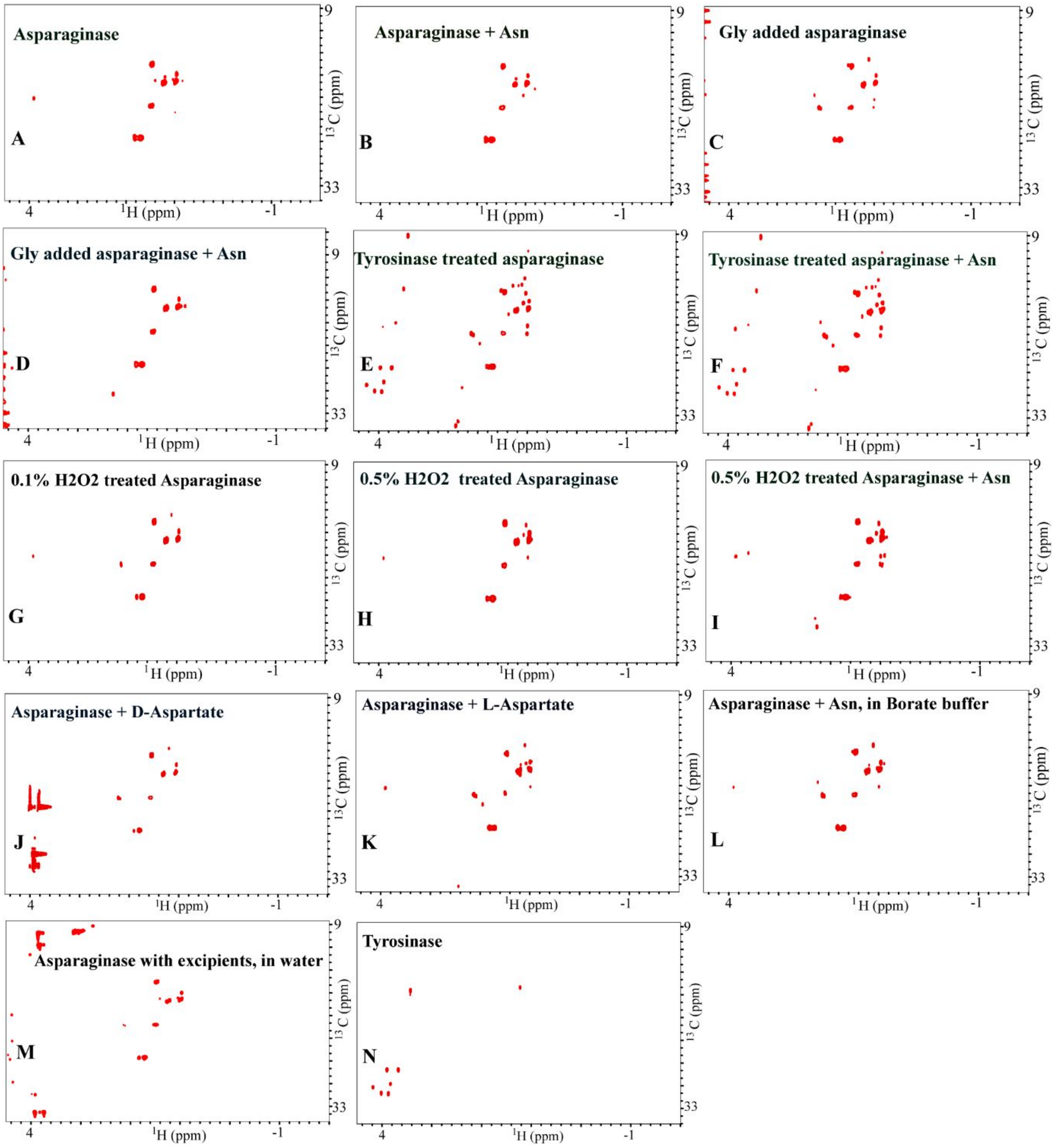

Figure S20. **[^{1}H-^{13}C] HSQC spectra of all the proteins used.**

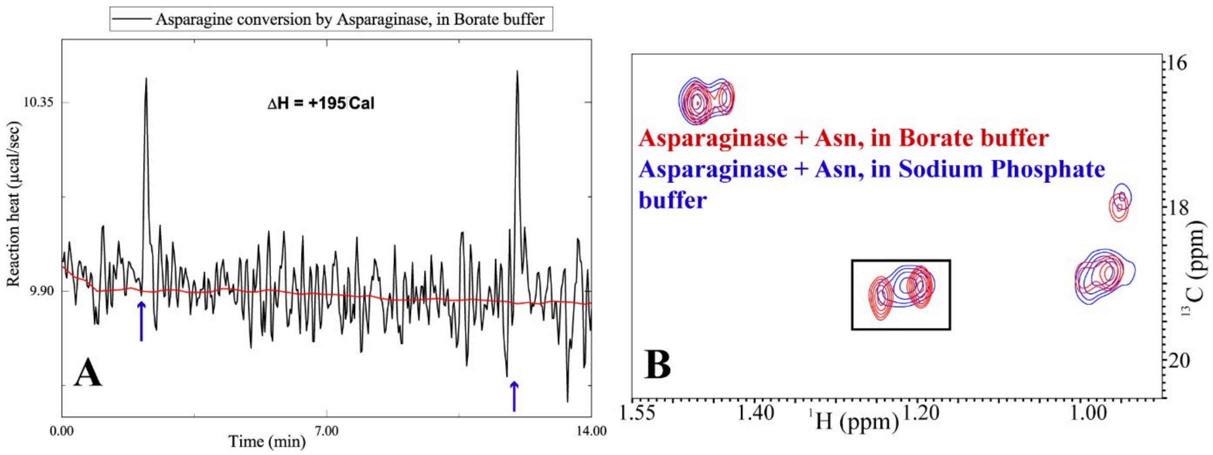

Figure S21. Asparaginase catalysis may not proceed in a borate buffer. (A) ITC thermogram showed positive apparent reaction enthalpy (+195Kcal) of Asn titration in borate buffer. This suggested that the reaction may not proceed in the borate buffer as the value is comparable to the asparagine dilution heat. **(B)** Methyl NMR spectra of Asparaginase in the presence and absence of asparagine failed to show any peak broadening for the critical loop residues, and borate buffer as determined before.

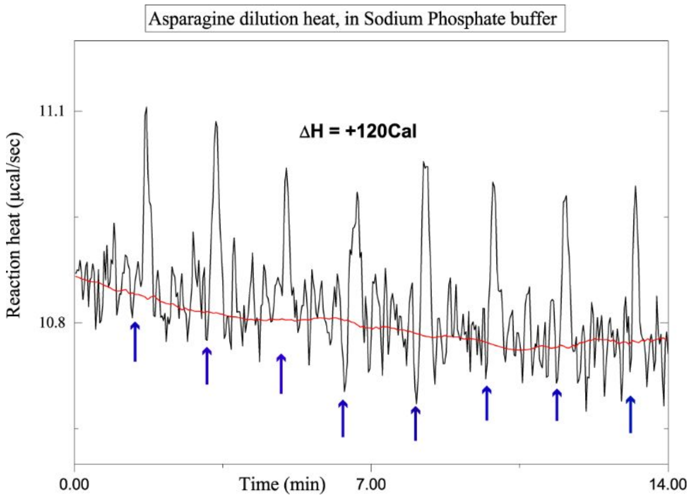

Figure S22: ITC thermogram showing positive apparent reaction enthalpy (+120Cal) upon addition of Asn in sodium phosphate buffer, pH 7.8. This is the dilution heat of Asn in the reaction buffer.
